# Prognostic value of body composition on early recurrence and long-term survival of resectable pancreatic ductal adenocarcinoma

**DOI:** 10.1007/s00330-025-12028-8

**Published:** 2025-11-10

**Authors:** Linxia Wu, Tong Nie, Xiaoling Zhi, Die Ouyang, Licai Zhang, Hongying Wu, Xin Li, Heshui Wu, Ping Han, Lei Chen, Feihong Wu, Chuansheng Zheng

**Affiliations:** 1 Department of Radiology, Union Hospital, Tongji Medical College, Huazhong University of Science and Technology, 1277 Jiefang Avenue, Wuhan, 430022 Hubei Province China; 2 Hubei Provincial Clinical Research Center for Precision Radiology & Interventional Medicine, Wuhan, 430022 Hubei Province China; 3 Hubei Key Laboratory of Molecular Imaging, Wuhan, 430022 Hubei Province China; 4 Department of Pancreatic Surgery, Union Hospital, Tongji Medical College, Huazhong University of Science and Technology, 1277 Jiefang Avenue, Wuhan, 430022 Hubei Province China

**Keywords:** Pancreas, Carcinoma (pancreatic ductal), Tomography (x-ray computed), Body composition, Recurrence

## Abstract

**Objective:**

To explore the associations of CT-evaluated body composition with early recurrence (ER) and overall survival (OS) in patients with pancreatic ductal adenocarcinoma (PDAC) after resection.

**Materials and methods:**

A retrospective analysis was performed on consecutively enrolled patients who underwent surgical resection for PDAC between 2014 and 2023. Patients were divided into a training set (*n* = 412) and a validation set (*n* = 120). Sex-specific cutoff values were determined for body composition indices, including visceral-to-subcutaneous fat ratio (VSR), skeletal muscle density (SMD), subcutaneous fat area (SFA), and other metrics. Logistic regression and Cox proportional hazards models were used to analyze the associations of body composition with ER and OS. Subgroup analyses were conducted based on clinicopathological characteristics to explore the prognostic value of body composition.

**Results:**

High VSR was an independent predictor for ER (OR: 2.304, *p *= 0.001) and worse OS (HR: 1.462, *p* = 0.007), whereas high SMD (HR: 0.609, *p* = 0.005) and high SFA (HR: 0.649, *p* = 0.002) were independent predictors for better OS. Subgroup analyses revealed variations in the prognostic effect of VSR according to diabetes status and tumor size. A model combining body composition metrics and clinicopathological indicators (carbohydrate antigen 19-9, carbohydrate antigen 12-5, tumor-node-metastasis stage, lymphovascular invasion, and adjuvant therapy) demonstrated good predictive ability for ER, with AUCs of 0.80 in the training set and 0.82 in the validation set.

**Conclusion:**

High VSR was an independent predictor for ER and worse OS in PDAC. Moreover, combining body composition metrics and clinicopathological indicators can improve the prognosis prediction of patients with PDAC after surgery.

**Key Points:**

***Question*** *What are the associations of CT-evaluated body composition with early recurrence and overall survival in patients with pancreatic ductal adenocarcinoma after resection?*

***Findings*** *High visceral-to-subcutaneous fat ratio is an independent predictor for early recurrence, whereas high skeletal muscle density and subcutaneous fat area independently predict better overall survival*.

***Clinical relevance*** *Combining body composition and clinicopathological indicators (carbohydrate antigen 19-9, carbohydrate antigen 12-5, tumor-node-metastasis stage, lymphovascular invasion, and adjuvant therapy) enables reliable prediction of postoperative prognosis in pancreatic ductal adenocarcinoma patients*.

**Graphical Abstract:**

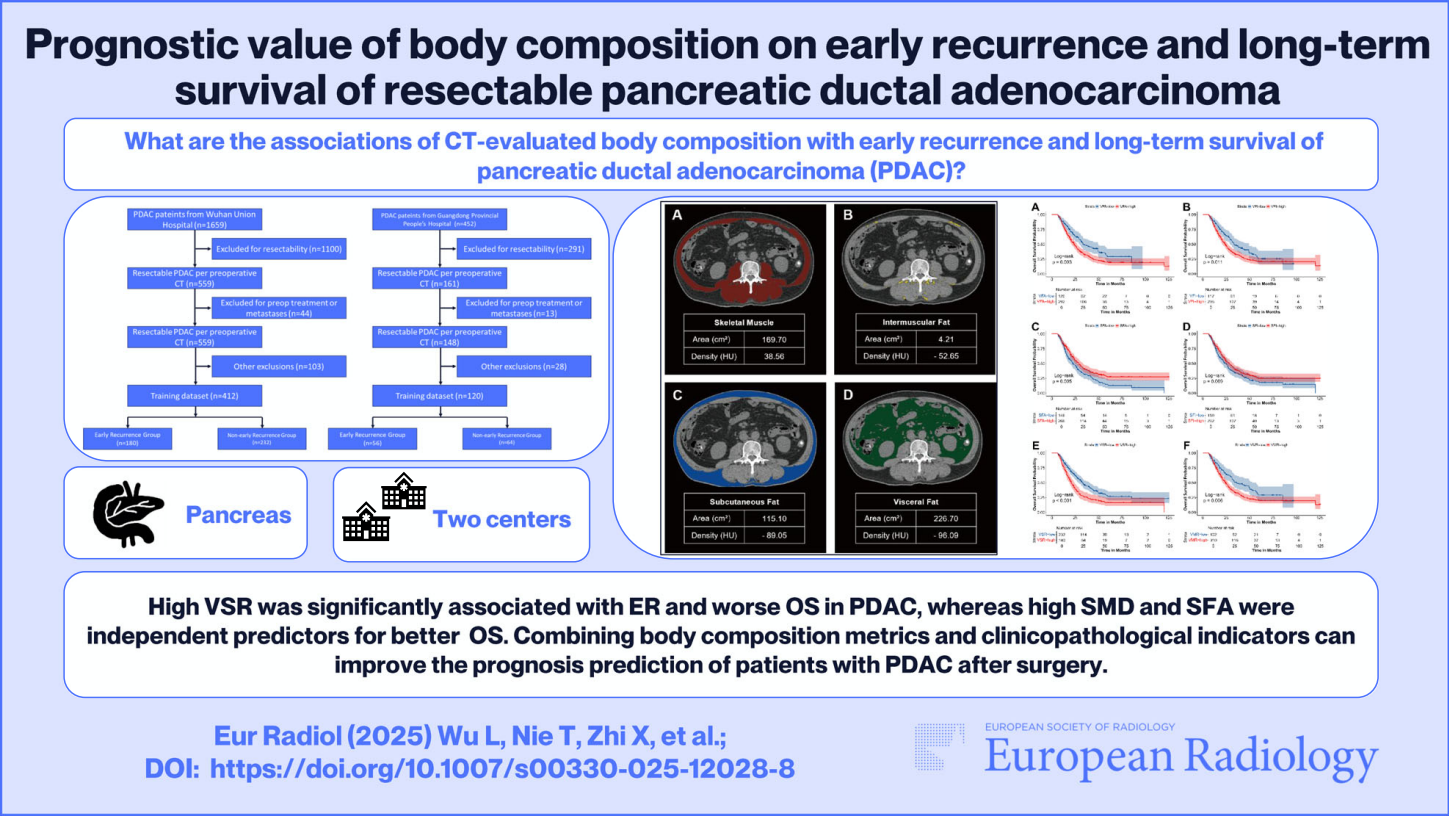

## Introduction

Pancreatic ductal adenocarcinoma (PDAC) is one of the most aggressive malignancies and a leading cause of cancer-related deaths worldwide [1]. Despite advancements in surgical techniques, treatments, and perioperative management, the prognosis of PDAC remains poor, with a 5-year survival rate below 20% due to the high incidence of tumor recurrence [2]. Tumor relapse occurs in up to 80% of patients within 5 years after surgical resection [3], with approximately 50% developing local progression or distant metastasis within the first year, termed early recurrence (ER) [4]. Patients with ER have a significantly worse prognosis, highlighting the critical importance of early intervention and personalized treatment strategies [5]. Identifying reliable indicators of ER in patients with PDAC and implementing individualized therapeutic strategies are essential for improving outcomes.

An increasing number of studies have shown that, in addition to tumor characteristics such as tumor size, differentiation, and tumor markers, body composition is also significantly associated with recurrence and long-term survival in PDAC [6–8]. Some changes in body composition are often related to tumor-associated metabolic alterations, malnutrition, inflammation, and muscle wasting [9–11]. In particular, reduced skeletal muscle quality and the accumulation of visceral fat have been strongly linked to poor prognosis in patients with PDAC [7, 8]. Such parameters are rarely assessed in routine clinical practice, even though they can be easily obtained from imaging examinations.

CT, known for its high-resolution and noninvasive nature, is the primary imaging modality for PDAC diagnosis and assessment [12]. Additionally, it enables precise quantification of body composition, including skeletal muscle and fat tissue [13, 14]. This study aimed to explore the relationship between CT-quantified body composition parameters, ER, and long-term survival in patients with PDAC. We also evaluated the potential value of these indicators in predicting ER, providing a basis for individualized surveillance, prevention, and treatment strategies.

## Materials and methods

### Study population

**Fig. 1:**
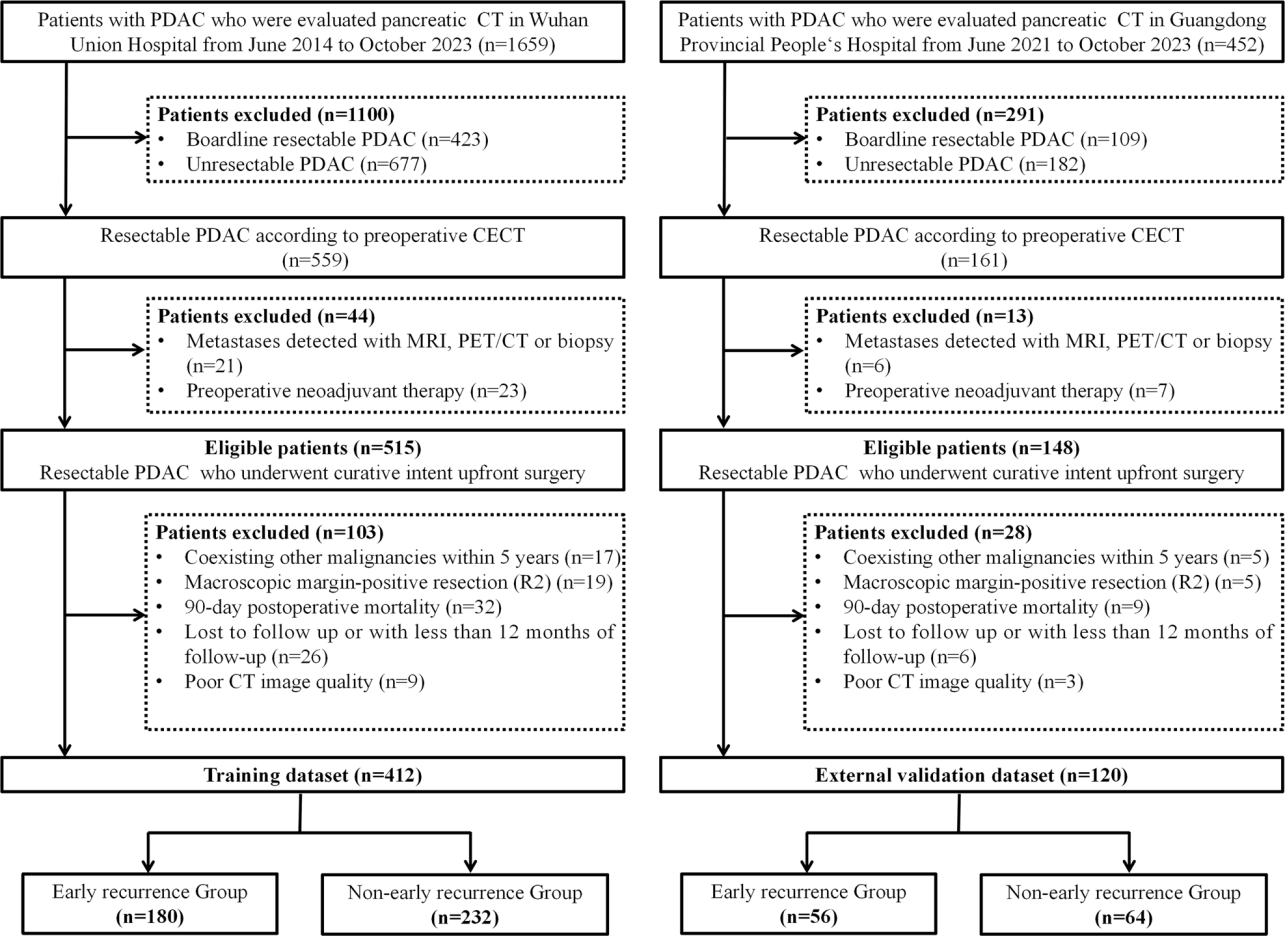
Flow diagram of study participants

We retrospectively and consecutively enrolled patients with resectable PDAC who underwent upfront surgery at Wuhan Union Hospital (June 2014—October 2023) as a training set. An external validation set was constructed by screening PDAC patients with the same criteria from Guangdong Provincial People’s Hospital (June 2021—October 2023). The inclusion criteria were as follows: (1) pathologically confirmed PDAC, (2) resectable disease determined by pancreatic CT (supplementary methods), and (3) CT examination performed within 2 weeks prior to surgery. The exclusion criteria were as follows: (1) macroscopic margin-positive resection (R2), (2) preoperative neoadjuvant therapy before surgery, (3) synchronous distant metastases at the time of surgery, (4) coexisting malignancy within 5 years before PDAC diagnosis, (5) 90-day postoperative mortality, (6) loss to follow-up or follow-up of less than 12 months, (7) incomplete clinical data, and (8) poor-quality CT images (Fig. 1). Informed consent was obtained from all patients, and the study was approved by the Medical Ethics Committee of each participating hospital.

### Data collection

Demographic characteristics and medical histories were extracted from electronic medical records. Tumor markers, including carbohydrate antigen (CA) 19-9 level, CA 12-5 level, carcinoembryonic antigen (CEA) level, white blood cell count, and platelet count, were collected using the measurements closest to the surgery and taken within 2 weeks preoperatively. Inflammation-based prognostic indexes, including platelet-lymphocyte ratio, neutrophil-lymphocyte ratio, lymphocyte-monocyte ratio, and prognostic nutrition index (PNI), calculated as serum albumin (g/L) + 5× total lymphocyte count (10^9^/L) [15]. Tumor location and size were measured using preoperative CT scans, and the presence of concurrent pancreatitis at diagnosis was confirmed through CT, clinical, and laboratory examinations. Pathologic findings from surgical specimens were extracted from electronic medical records, including resection margin status (R0/R1), tumor-node-metastasis (TNM) staging according to the eighth edition of the American Joint Committee on Cancer staging system [16], and details of tumor differentiation, perineural invasion, and lymphovascular involvement.

### Follow-up and definitions of study endpoint

Postoperative surveillance included contrast-enhanced CT after resection and serum CA 19-9 measurements every 3 months during the first 2 years postoperatively and every 6 months thereafter at each institute. When imaging features suggested potential cancer recurrence, additional magnetic resonance imaging (MRI) and/or fluorodeoxyglucose positron emission tomography (PET) scans would be performed to further evaluate ambiguous CT findings. Tumor recurrence was defined as a relapse confirmed by either radiological or pathological evidence. Radiological recurrence was characterized by the detection of new lesions in anatomical sites, including the surgical bed, lungs, liver, and peritoneum [17]. Pathological confirmation via biopsy was only performed when radiological findings were equivocal. The primary endpoint was ER, defined as tumor relapse within 1 year after curative resection [4]. The secondary endpoint was overall survival (OS), defined as the time from the initial surgery to death or the last follow-up. The study’s final follow-up date was October 1, 2024.

### Body composition analysis

**Fig. 2:**
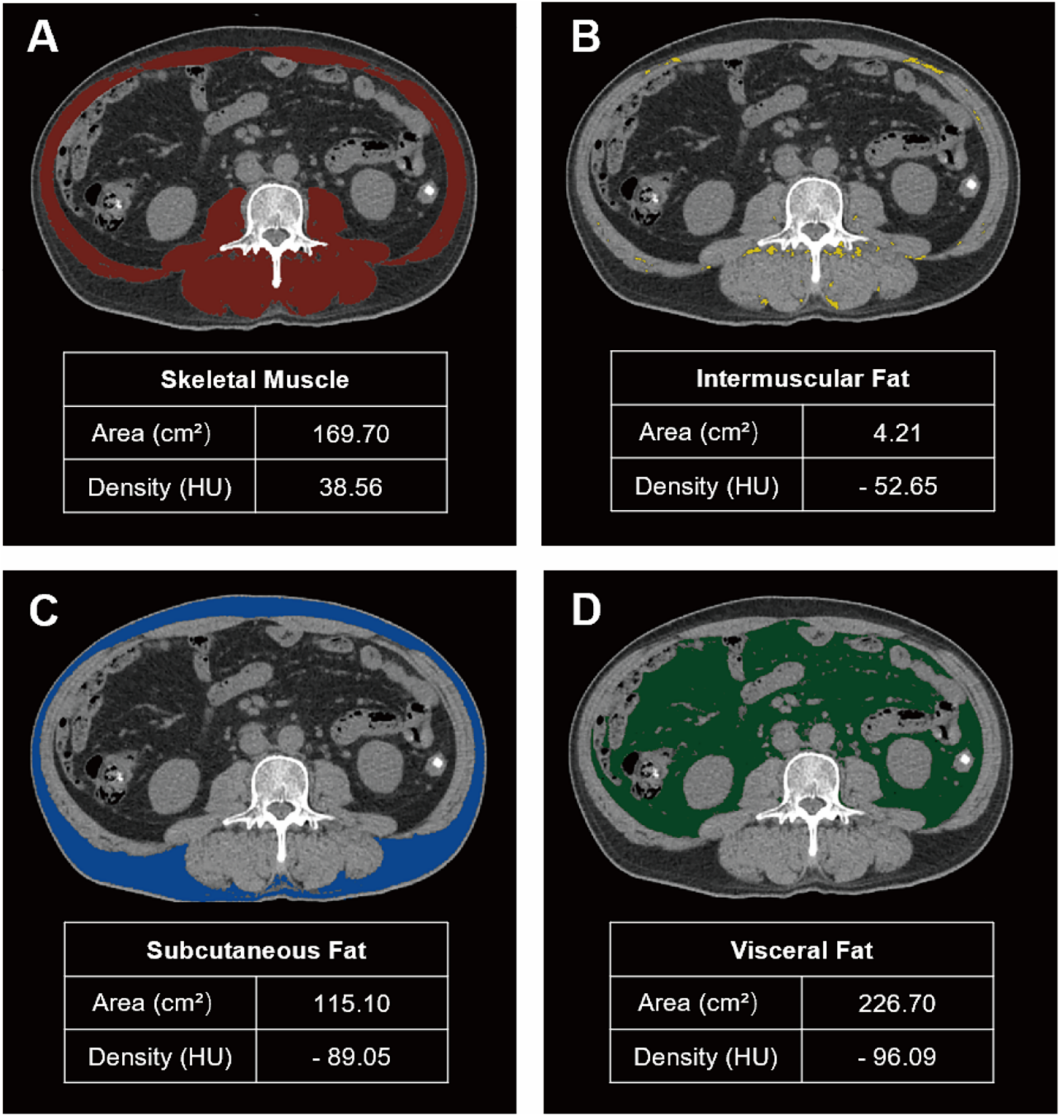
Measurement of body composition variables. **A** The areas of total skeletal muscle were measured with tissue Hounsfield unit (HU) thresholds from −29 to +150 HU. **B** The areas of intermuscular fat area were measured with tissue Hounsfield unit (HU) thresholds from −190 to −30 HU. **C** The areas of subcutaneous fat were measured with tissue HU thresholds from −190 to −30 HU. **D** The areas of visceral fat were measured with tissue HU thresholds from −150 to −50 HU.

Abdominal CT images of patients with PDAC were acquired from the institutional PACS system within 2 weeks prior to surgery. CT scanning parameters were as follows: tube voltage of 90–120 kVp, tube current of 100–300 mAs, and slice thickness of 1–5 mm. Body composition parameters were measured from transverse non-enhanced CT images at the third lumbar vertebra (L3) level using SliceOMatic V5.0 (Fig. 2). The subcutaneous fat area (SFA), visceral fat area (VFA), skeletal muscle area (SMA), and intermuscular fat area (IMFA) were measured using tissue-specific Hounsfield unit (HU) ranges: −29 to +150 HU for SMA, −150 to −50 HU for VFA, and −190 to −30 HU for both SFA and IMFA. These areas were normalized to the patient’s height squared and expressed as skeletal muscle index (SMI), visceral fat index (VFI), subcutaneous fat index (SFI), and intermuscular fat index (IMFI).

Skeletal muscle density (SMD) index, also known as muscle radiodensity, serves as a proxy for muscle quality. The visceral-to-subcutaneous fat tissue area ratio (VSR) was calculated as VFA (cm^2^)/SFA (cm^2^) and regarded as a proxy for visceral fat accumulation [7]. The visceral-to-muscle ratio (VMR) index (VFA (cm^2^)/SMA (cm^2^)) was used as a proxy for sarcopenic obesity [8]. Inter-observer reliability of these measurements was assessed between two radiologists with five (L.W.) and three (T.N.) years of experience in musculoskeletal imaging, using intraclass correlation coefficients (ICCs) and Bland-Altman analysis. ICCs were categorized as poor (< 0.40), fair (0.40–0.59), good (0.60–0.74), or excellent (0.75–1.00). Mean values from both observers were used in the final analysis.

### Statistical analysis

All statistical analyses were performed using R version 4.3.3 (https://www.r-project.org). Continuous numerical variables were presented as mean ± standard deviations (SD) or median and interquartile range (IQR), whereas categorical variables were reported as numbers (N) and percentages. The chi-squared test or Fisher’s exact test was used to analyze categorical variables, with the Student’s *t*-test or Mann–Whitney *U*-test for continuous variables. To account for multiple testing, two-sided *p*-values were adjusted using the Benjamini–Hochberg (B-H) method to control the false discovery rate (FDR). An association was considered statistically significant if its corresponding B-H-adjusted *p*-value was below 0.05, corresponding to an FDR threshold of 5%. Optimal cut-off values for each body composition parameter were determined separately by sex using the “surv_cutpoint” function from the “survminer” R package, which identifies optimal thresholds based on survival analysis by maximizing the log-rank test statistic. Patients were then grouped accordingly. Similarly, the cut-off values for age, body mass index (BMI), tumor size, CA 19-9, CA 12-5, CEA, albumin, platelet-lymphocyte ratio, neutrophil-lymphocyte ratio, lymphocyte-monocyte ratio, and PNI were calculated using the same survival-based method (i.e., “surv_cutpoint”).

Associations between potential risk factors and early PDAC recurrence were assessed using univariable and multivariable logistic regression analyses. Survival analyses were performed using the Kaplan–Meier method and compared using the log-rank test. Univariable and multivariable Cox proportional hazard regression analyses were performed to identify risk factors for OS. A *p*-value of < 0.05 was considered statistically significant. Subsequently, based on the optimal cut-off values determined in the training set, we validated the model using an external dataset.

## Results

### Patients

The training set comprised 412 patients (with 201 aged > 60 years (48.79%); 249 men (60.44%)). Furthermore, the external validation set consisted of 120 patients (with 66 aged > 60 years (55.0%); 70 men (58.33%)). As detailed in Table S1, baseline clinicopathological characteristics were comparable between groups, except for median follow-up duration (training set: 50.7 months (IQR: 44.7–54.8 months) vs. external validation set: 26.4 months (IQR: 23.6–27.9 months); *p* < 0.001). Table 1 provided detailed information on the general clinical characteristics of the patients in the training set. Early recurrence occurred in 180 patients (43.68%), who had a median OS of 14.4 months (95% CI: 13.3–16.0 months) and were categorized as the ER group, and the remaining 232 patients (56.32%) with a median OS of 44.6 months (95% CI: 39.2–59.1 months) were categorized as the non-ER group. Body composition parameters were divided into low and high groups based on pre-defined cut-offs (Table S2). Good inter-observer agreement was observed between the two radiologists for body composition measurements, with all ICC values exceeding 0.95, along with minimal mean differences and narrow limits of agreement (Table S3 and Fig. S1).

**Table 1:** Patient characteristics between the early recurrence and non-early recurrence groups in the training set

Characteristics	Total (*n* = 412)	Non-early recurrence (*n* = 232)	Early recurrence (*n* = 180)	*p-*value	Adjusted *p*-value
Age (years)				0.92	0.95
≤ 60	211 (51.21)	119 (51.29)	92 (51.11)		
> 60	201 (48.79)	113 (48.71)	88 (48.89)		
Gender				0.08	0.15
Female	163 (39.56)	101 (43.53)	62 (34.44)		
Male	249 (60.44)	131 (56.47)	118 (65.56)		
BMI (kg/m^2^)				0.65	0.81
≤ 23.8	294 (71.36)	163 (70.26)	131 (72.78)		
> 23.8	118 (28.64)	69 (29.74)	49 (27.22)		
Smoking history				0.14	0.22
No	287 (69.66)	169 (72.84)	118 (65.56)		
Yes	125 (30.34)	63 (27.16)	62 (34.44)		
Diabetes history				0.4	0.58
No	325 (78.88)	187 (80.6)	138 (76.67)		
Yes	87 (21.12)	45 (19.4)	42 (23.33)		
Pancreatitis at diagnosis				0.13	0.22
No	373 (90.53)	215 (92.67)	158 (87.78)		
Yes	39 (9.47)	17 (7.33)	22 (12.22)		
Tumor Location				0.94	0.95
Head or neck	311 (75.49)	175 (75.43)	136 (75.56)		
Body or tail	101 (24.51)	57 (24.57)	44 (24.44)		
Tumor Size (cm)				**< 0.001**	**< 0.001**
≤ 2.9	215 (52.18)	145 (62.5)	70 (38.89)		
> 2.9	197 (47.82)	87 (37.5)	110 (61.11)		
CA 19-9 (U/mL)				**< 0.001**	**< 0.001**
≤ 538.4	278 (67.48)	186 (80.17)	92 (51.11)		
> 538.4	134 (32.52)	46 (19.83)	88 (48.89)		
CA 12-5 (U/mL)				**< 0.001**	**< 0.001**
≤ 14.2	159 (38.59)	114 (49.14)	45 (25.0)		
> 14.2	253 (61.41)	118 (50.86)	135 (75.0)		
CEA (ug/L)				0.06	0.13
≤ 9.6	361 (87.62)	210 (90.52)	151 (83.89)		
> 9.6	51 (12.38)	22 (9.48)	29 (16.11)		
Albumin (g/L)				**0.04**	0.09
≤ 38.7	248 (60.19)	129 (55.6)	119 (66.11)		
> 38.7	164 (39.81)	103 (44.4)	61 (33.89)		
PLR				0.47	0.62
≤ 193.0	306 (74.27)	176 (75.86)	130 (72.22)		
> 193.0	106 (25.73)	56 (24.14)	50 (27.78)		
NLR				0.2	0.3
≤ 1.6	72 (17.48)	46 (19.83)	26 (14.44)		
> 1.6	340 (82.52)	186 (80.17)	154 (85.56)		
LMR				0.4	0.58
≤ 5.5	361 (87.62)	200 (86.21)	161 (89.44)		
> 5.5	51 (12.38)	32 (13.79)	19 (10.56)		
PNI				**0.01**	**0.03**
≤ 42.7	149 (36.17)	71 (30.6)	78 (43.33)		
> 42.7	263 (63.83)	161 (69.4)	102 (56.67)		
Margin				**0.02**	0.06
R0	352 (85.44)	207 (89.22)	145 (80.56)		
R1	60 (14.56)	25 (10.78)	35 (19.44)		
Lymphovascular invasion				**< 0.001**	**< 0.001**
Absent	246 (59.71)	171 (73.71)	75 (41.67)		
Present	166 (40.29)	61 (26.29)	105 (58.33)		
Nerve infiltration				**0.04**	0.09
Absent	21 (5.1)	17 (7.33)	4 (2.22)		
Present	391 (94.9)	215 (92.67)	176 (97.78)		
Grade				**0.007**	**0.03**
Well or moderate	286 (69.42)	174 (75.0)	112 (62.22)		
Poor	126 (30.58)	58 (25.0)	68 (37.78)		
T stage				0.1	0.18
T1	84 (20.39)	56 (24.14)	28 (15.56)		
T2	237 (57.52)	128 (55.17)	109 (60.56)		
T3	91 (22.09)	48 (20.69)	43 (23.89)		
N stage				**< 0.001**	**< 0.001**
N0	195 (47.33)	137 (59.05)	58 (32.22)		
N1	163 (39.56)	75 (32.33)	88 (48.89)		
N2	54 (13.11)	20 (8.62)	34 (18.89)		
TNM				**< 0.001**	**< 0.001**
≤ IIA	199 (48.3)	135 (58.19)	64 (35.56)		
≥ IIB	213 (51.7)	97 (41.81)	116 (64.44)		
Adjuvant				**< 0.001**	**< 0.001**
No	81 (19.66)	29 (12.5)	52 (28.89)		
Yes	331 (80.34)	203 (87.5)	128 (71.11)		
VFA (cm^2^)				0.05	0.11
Low	120 (29.13)	77 (33.19)	43 (23.89)		
High	292 (70.87)	155 (66.81)	137 (76.11)		
VFI (cm^2^/m^2^)				**0.03**	0.09
Low	117 (28.4)	76 (32.76)	41 (22.78)		
High	295 (71.6)	156 (67.24)	139 (77.22)		
SFA (cm^2^)				0.57	0.74
Low	146 (35.44)	79 (34.05)	67 (37.22)		
high	266 (64.56)	153 (65.95)	113 (62.78)		
SFI (cm^2^/m^2^)				0.84	0.95
Low	150 (36.41)	83 (35.78)	67 (37.22)		
High	262 (63.59)	149 (64.22)	113 (62.78)		
SMA (cm^2^)				0.72	0.84
Low	242 (58.74)	134 (57.76)	108 (60.0)		
High	170 (41.26)	98 (42.24)	72 (40.0)		
SMI (cm^2^/m^2^)				0.93	0.95
Low	278 (67.48)	157 (67.67)	121 (67.22)		
High	134 (32.52)	75 (32.33)	59 (32.78)		
IMFA (cm^2^)				0.72	0.84
Low	121 (29.37)	66 (28.45)	55 (30.56)		
High	291 (70.63)	166 (71.55)	125 (69.44)		
IMFI (cm^2^/m^2^)				0.42	0.58
low	230 (55.83)	125 (53.88)	105 (58.33)		
High	182 (44.17)	107 (46.12)	75 (41.67)		
SMD (HU)				0.95	0.95
Low	67 (16.26)	37 (15.95)	30 (16.67)		
High	345 (83.74)	195 (84.05)	150 (83.33)		
VSR				**< 0.001**	**< 0.001**
Low	232 (56.31)	155 (66.81)	77 (42.78)		
High	180 (43.69)	77 (33.19)	103 (57.22)		
VMR				0.06	0.13
Low	102 (24.76)	66 (28.45)	36 (20.0)		
High	310 (75.24)	166 (71.55)	144 (80.0)		
Median overall survival	26.2 (23.7, 29.4)	44.6 (39.2, 59.1)	14.4 (13.3, 16.0)	**< 0.001**	**< 0.001**

Data are presented as *n* (%)

Adjusted *p*-values were computed using the Benjamini–Hochberg method for false discovery rate control

Bold was used to highlight values that were statistically significant (*p*-value < 0.05)

*BMI* body mass index, *CA 19-9* carbohydrate antigen 19-9, *CA 12-5* carbohydrate antigen 12-5, *CEA* carcinoembryonic antigen, *LMR* lymphocyte-to-monocyte ratio, *NLR* neutrophil-to-lymphocyte ratio, *PLR* platelet-to-lymphocyte ratio, *PNI* prognostic nutritional index, *R0* negative surgical margin, *R1* positive surgical margin, *IMFA* intermuscular fat area, *IMFI* intermuscular fat index, *SFA* subcutaneous fat area, *SFI* subcutaneous fat index, *SMA* skeletal muscle area, *SMD* skeletal muscle density, *SMI* skeletal muscle index, *TNM* tumor-node-metastasis, *VFA* visceral fat area, *VFI* visceral fat index; *VSR* VFA-to-SFA ratio, *VMR* VFA-to-SMA ratio

The ER group in the training set had a significantly higher proportion of patients with larger tumor size (> 2.9 cm: 61.11% vs. 37.5%, adjusted *p* < 0.001), elevated CA 19-9 (> 538.4 U/L: 48.89% vs. 19.83%, adjusted *p* < 0.001), higher CA 12-5 (> 14.2 U/L: 75.0% vs. 50.86%, adjusted *p* < 0.001), and lower PNI (≤ 42.7: 43.33% vs. 30.6%, adjusted *p* = 0.03) (Table 1). Regarding pathological indicators, the ER group had a higher proportion of patients with lymphovascular invasion (58.33% vs. 26.29%, adjusted *p* < 0.001), poorly differentiated tumors (37.78% vs. 25.0%, adjusted *p* = 0.03), lymph node metastases (N1: 48.89% vs. 32.33%, N2: 18.89% vs. 8.62%, adjusted *p* < 0.001), and advanced TNM stage (≥ IIB) (64.44% vs. 41.81%, adjusted *p* < 0.001). Regarding body composition, patients in the ER group had higher VSR (57.22% vs. 33.19%, adjusted *p* < 0.001) than those in the non-ER group. While the ER group had a lower proportion of patients who received adjuvant therapy (71.11% vs. 87.5%, adjusted *p* < 0.001). Notably, the ER group in the training set had a higher proportion of patients with lower Albumin level (≤ 38.7 g/L: 66.11% vs. 55.6%, *p* = 0.04, adjusted *p* = 0.09), positive resection margin (R1: 19.44% vs. 10.78%, *p* = 0.02, adjusted *p* = 0.06), nerve infiltration (97.78% vs. 92.67%, *p* = 0.04, adjusted *p* = 0.09) and higher VFI (77.22% vs. 67.24%, *p* = 0.03, adjusted *p* = 0.09) compared to the non-ER group. However, after correction for multiple testing, these differences did not reach statistical significance.

### Clinicopathological characteristics and body composition-based VSR

Table 2 presented the clinicopathological characteristics and body composition of the high and low VSR groups in the training set. More patients in the high-VSR group were men (85.56% vs. 40.95%, adjusted *p* < 0.001) and had a history of smoking (44.44% vs. 19.4%, adjusted *p* < 0.001), as well as diabetes (27.22% vs. 16.38%, adjusted *p* = 0.03), a high BMI level (35.0% vs. 23.71%, adjusted *p* = 0.04), a high CA 19-9 level (43.89% vs. 23.71%, adjusted *p* < 0.001), a positive resection margin (19.44% vs. 10.78%, adjusted *p* = 0.045), a poor tumor grade (37.22% vs. 25.43%, adjusted *p* = 0.04), and a high risk of ER (57.22% vs. 33.19%, adjusted *p* < 0.001) compared to the low VSR group. Body composition analysis revealed that the high-VSR group exhibited higher VFA, VFI, SFA, SFI, IMFA, and VMR, but lower SMA (all adjusted *p* < 0.05). It was worth noting that the high-VSR group in the training set had a higher proportion of patients with elevated CA 12-5 level (> 14.2 U/mL: 67.78% vs. 56.47%, *p *= 0.03) compared to the low VSR group, though this difference did not reach statistical significance after correction for multiple testing (adjusted *p* = 0.06).

**Table 2:** Patient characteristics between the VSR-high and VSR-low groups in the training set

Characteristics	Total (*n* = 412)	VSR-low (*n* = 232)	VSR-high (*n* = 180)	*p*-value	Adjusted *p*-value
Age (years)				0.74	0.89
≤ 60	211 (51.21)	121 (52.16)	90 (50.0)		
> 60	201 (48.79)	111 (47.84)	90 (50.0)		
Gender				**< 0.001**	**< 0.001**
Female	163 (39.56)	137 (59.05)	26 (14.44)		
Male	249 (60.44)	95 (40.95)	154 (85.56)		
BMI (kg/m^2^)				**0.02**	**0.04**
≤ 23.8	294 (71.36)	177 (76.29)	117 (65.0)		
> 23.8	118 (28.64)	55 (23.71)	63 (35.0)		
Smoking history				**< 0.001**	**< 0.001**
No	287 (69.66)	187 (80.6)	100 (55.56)		
Yes	125 (30.34)	45 (19.4)	80 (44.44)		
Diabetes history				**0.01**	**0.03**
No	325 (78.88)	194 (83.62)	131 (72.78)		
Yes	87 (21.12)	38 (16.38)	49 (27.22)		
Pancreatitis at diagnosis				0.88	0.96
No	373 (90.53)	211 (90.95)	162 (90.0)		
Yes	39 (9.47)	21 (9.05)	18 (10.0)		
Tumor Location				0.44	0.66
Head or neck	311 (75.49)	179 (77.16)	132 (73.33)		
Body or tail	197 (47.82)	53 (22.84)	48 (26.67)		
Tumor Size (cm)				0.5	0.7
≤ 2.9	215 (52.18)	125 (53.88)	90 (50.0)		
> 2.9	101 (24.51)	107 (46.12)	90 (50.0)		
CA 19-9 (U/mL)				**< 0.001**	**< 0.001**
≤ 538.4	278 (67.48)	177 (76.29)	101 (56.11)		
> 538.4	134 (32.52)	55 (23.71)	79 (43.89)		
CA 12-5 (U/mL)				**0.03**	0.06
≤ 14.2	159 (38.59)	101 (43.53)	58 (32.22)		
> 14.2	253 (61.41)	131 (56.47)	122 (67.78)		
CEA (ug/L)				0.2	0.4
≤ 9.6	361 (87.62)	208 (89.66)	153 (85.0)		
> 9.6	51 (12.38)	24 (10.34)	27 (15.0)		
Albumin (g/L)				1	1
≤ 38.7	248 (60.19)	140 (60.34)	108 (60.0)		
> 38.7	164 (39.81)	92 (39.66)	72 (40.0)		
PLR				0.52	0.7
≤ 193.0	306 (74.27)	169 (72.84)	137 (76.11)		
> 193.0	106 (25.73)	63 (27.16)	43 (23.89)		
NLR				0.79	0.92
≤ 1.6	72 (17.48)	39 (16.81)	33 (18.33)		
> 1.6	340 (82.52)	193 (83.19)	147 (81.67)		
LMR				0.59	0.74
≤ 5.5	361 (87.62)	201 (86.64)	160 (88.89)		
> 5.5	51 (12.38)	31 (13.36)	20 (11.11)		
PNI				0.59	0.74
≤ 42.7	149 (36.17)	87 (37.5)	62 (34.44)		
> 42.7	263 (63.83)	145 (62.5)	118 (65.56)		
Margin				**0.02**	**0.045**
R0	352 (85.44)	207 (89.22)	145 (80.56)		
R1	60 (14.56)	25 (10.78)	35 (19.44)		
Lymphovascular invasion				0.11	0.22
Absent	246 (59.71)	147 (63.36)	99 (55.0)		
Present	166 (40.29)	85 (36.64)	81 (45.0)		
Nerve infiltration				0.45	0.66
Absent	21 (5.1)	14 (6.03)	7 (3.89)		
Present	391 (94.9)	218 (93.97)	173 (96.11)		
Grade				**0.01**	**0.04**
Well or moderate	286 (69.42)	173 (74.57)	113 (62.78)		
Poor	126 (30.58)	59 (25.43)	67 (37.22)		
T stage				0.39	0.62
T1	84 (20.39)	52 (22.41)	32 (17.78)		
T2	237 (57.52)	133 (57.33)	104 (57.78)		
T3	91 (22.09)	47 (20.26)	44 (24.44)		
N stage				0.34	0.59
N0	195 (47.33)	110 (47.41)	85 (47.22)		
N1	163 (39.56)	87 (37.5)	76 (42.22)		
N2	54 (13.11)	35 (15.09)	19 (10.56)		
TNM				0.93	0.99
≤ IIA	199 (48.3)	113 (48.71)	86 (47.78)		
≥ IIB	213 (51.7)	119 (51.29)	94 (52.22)		
Adjuvant				0.96	0.99
No	81 (19.66)	46 (19.83)	35 (19.44)		
Yes	331 (80.34)	186 (80.17)	145 (80.56)		
VFA (cm^2^)				**< 0.001**	**< 0.001**
Low	120 (29.13)	96 (41.38)	24 (13.33)		
High	292 (70.87)	136 (58.62)	156 (86.67)		
VFI (cm^2^/m^2^)				**< 0.001**	**< 0.001**
Low	117 (28.4)	90 (38.79)	27 (15.0)		
High	295 (71.6)	142 (61.21)	153 (85.0)		
SFA (cm^2^)				**< 0.001**	**0.002**
Low	146 (35.44)	99 (42.67)	47 (26.11)		
High	266 (64.56)	133 (57.33)	133 (73.89)		
SFI (cm^2^/m^2^)				**< 0.001**	**< 0.001**
Low	150 (36.41)	106 (45.69)	44 (24.44)		
High	262 (63.59)	126 (54.31)	136 (75.56)		
SMA (cm^2^)				**< 0.001**	**< 0.001**
Low	242 (58.74)	106 (45.69)	136 (75.56)		
High	170 (41.26)	126 (54.31)	44 (24.44)		
SMI (cm^2^/m^2^)				0.84	0.95
Low	278 (67.48)	158 (68.1)	120 (66.67)		
High	134 (32.52)	74 (31.9)	60 (33.33)		
IMFA (cm^2^)				**0.01**	**0.04**
Low	121 (29.37)	80 (34.48)	41 (22.78)		
High	291 (70.63)	152 (65.52)	139 (77.22)		
IMFI (cm^2^/m^2^)				0.32	0.58
Low	230 (55.83)	124 (53.45)	106 (58.89)		
High	182 (44.17)	108 (46.55)	74 (41.11)		
SMD (HU)				0.39	0.62
Low	67 (16.26)	34 (14.66)	33 (18.33)		
High	345 (83.74)	198 (85.34)	147 (81.67)		
VMR				**< 0.001**	**< 0.001**
Low	102 (24.76)	84 (36.21)	18 (10.0)		
High	310 (75.24)	148 (63.79)	162 (90.0)		
Early recurrence				**< 0.001**	**< 0.001**
No	232 (56.31)	155 (66.81)	77 (42.78)		
Yes	180 (43.69)	77 (33.19)	103 (57.22)		

Data are presented as *n* (%)

Adjusted *p*-values were computed using the Benjamini–Hochberg method for false discovery rate control

Bold was used to highlight values that were statistically significant (*p*-value < 0.05)

*BMI* body mass index, *CA 19-9* carbohydrate antigen 19-9, *CA 12-5* carbohydrate antigen 12-5, *CEA* carcinoembryonic antigen, *LMR* lymphocyte-to-monocyte ratio, *NLR* neutrophil-to-lymphocyte ratio, *PLR* platelet-to-lymphocyte ratio, *PNI* prognostic nutritional index, *R0* negative surgical margin, *R1* positive surgical margin, *IMFA* intermuscular fat area, *IMFI* intermuscular fat index, *SFA* subcutaneous fat area, *SFI* subcutaneous fat index, *SMA* skeletal muscle area, *SMD* skeletal muscle density, *SMI* skeletal muscle index, *TNM* tumor-node-metastasis, *VFA* visceral fat area, *VFI* visceral fat index, *VSR* VFA-to-SFA ratio, *VMR* VFA-to-SMA ratio

### Risk factors for early recurrence after PDAC resection

Univariable logistic regression analysis in the training set identified several factors associated with ER after PDAC resection, including CA 19-9, CA 12-5, CEA, PNI, resection margin, lymphovascular invasion, nerve infiltration, tumor grade, TNM stage, adjuvant therapy, VFA, VFI, and VSR. The multivariable analysis revealed the following independent risk factors for ER: high CA 19-9 level (> 538.4 U/L) (odds ratio (OR)  =  2.412, 95% CI: 1.438–4.043, *p*  =  0.001), high CA 12-5 level (> 14.2 U/L) (OR  =  1.845, 95% CI: 1.105–3.080, *p*  =  0.02), the presence of lymphovascular invasion (OR  =  2.719, 95% CI: 1.664–4.445, *p*  <  0.001), TNM stage ≥ IIB (OR  =  1.772, 95% CI: 1.092–2.875, *p*  =  0.02), and high VSR  (OR  =  2.304, 95% CI: 1.396–3.801, *p*  =  0.001) (Table 3). While postoperative adjuvant therapy (OR = 0.333, 95% CI: 0.182–0.610, *p* < 0.001) was identified as an independent protective factor, significantly reducing the risk of postoperative ER in patients with PDAC.

**Table 3:** Univariable and multivariable logistic regression of factors associated with early recurrence in the training set

Characteristics	Univariable analysis		Multivariable analysis	
	OR (95% CI)	*p*-value	OR (95% CI)	*p*-value
Age > 60 years	1.007 (0.682–1.487)	0.97		
Gender (Male)	1.467 (0.981–2.194)	0.06		
BMI > 23.8 kg/m^2^	0.884 (0.573–1.362)	0.58		
Smoking history	1.409 (0.924–2.150)	0.11		
Diabetes history	1.265 (0.787–2.033)	0.33		
Pancreatitis at diagnosis	1.761 (0.905–3.426)	0.1		
Tumor location (body or tail)	0.993 (0.632–1.562)	0.98		
Tumor size > 2.9 cm	0.892 (0.566–1.406)	0.62		
CA 19-9 > 538.4 U/L	3.868 (2.502–5.978)	**< 0.001**	2.412 (1.438–4.043)	**0.001**
CA 12-5 > 14.2 U/L	2.898 (1.896–4.430)	**< 0.001**	1.845 (1.105–3.080)	**0.02**
CEA > 9.6 ug/L	1.833 (1.014–3.315)	**0.045**	0.757 (0.368–1.556)	0.67
PLR > 193.0	1.209 (0.776–1.884)	0.4		
NLR > 1.6	1.465 (0.866–2.479)	0.16		
LMR > 5.5	0.738 (0.403–1.350)	0.32		
PNI > 42.7	0.577 (0.384–0.866)	**0.008**	0.711 (0.433–1.168)	0.18
Resection margin (R1)	1.999 (1.147–3.483)	**0.02**	1.207 (0.619–2.353)	0.58
Lymphovascular invasion	3.925 (2.588–5.951)	**< 0.001**	2.719 (1.664–4.445)	**< 0.001**
Nerve infiltration	3.479 (1.150–10.524)	**0.03**	1.429 (0.397–5.146)	0.59
Grade (Poor)	1.821 (1.193–2.781)	**0.005**	1.327 (0.785–2.242)	0.29
TNM (≥ IIB)	2.523 (1.688–3.769)	**< 0.001**	1.772 (1.092–2.875)	**0.02**
Adjuvant therapy	0.352 (0.212–0.583)	**< 0.001**	0.333 (0.182–0.610)	**< 0.001**
VFA (high)	1.583 (1.021–2.453)	**0.04**	0.692 (0.169–2.834)	0.61
VFI (high)	1.652 (1.06–2.573)	**0.03**	1.987 (0.483–8.180)	0.34
SFA (high)	0.871 (0.580–1.307)	0.51		
SFI (high)	0.939 (0.627–1.407)	0.76		
SMA (high)	0.912 (0.614–1.354)	0.65		
SMI (high)	1.021 (0.674–1.546)	0.92		
IMFA (high)	0.904 (0.590–1.384)	0.64		
IMFI (high)	0.834 (0.563–1.236)	0.37		
SMD (high)	0.949 (0.560–1.606)	0.85		
VSR (high)	2.693 (1.801–4.026)	**< 0.001**	2.304 (1.396–3.801)	**0.001**
VMR (high)	1.59 (1.001–2.528)	0.05		

Bold was used to highlight values that were statistically significant (*p*-value < 0.05)

*BMI* body mass index, *CA 19-9* carbohydrate antigen 19-9, *CA 12-5* carbohydrate antigen 12-5, *CEA* carcinoembryonic antigen, *LMR* lymphocyte-to-monocyte ratio, *NLR* neutrophil-to-lymphocyte ratio, *PLR* platelet-to-lymphocyte ratio, *PNI* prognostic nutritional index, *R0* negative surgical margin, *R1* positive surgical margin, *IMFA* intermuscular fat area, *IMFI* intermuscular fat index, *SFA* subcutaneous fat area, *SFI* subcutaneous fat index, *SMA* skeletal muscle area, *SMD* skeletal muscle density, *SMI* skeletal muscle index, *TNM* tumor-node-metastasis, *VFA* visceral fat area, *VFI* visceral fat index,* VSR* VFA-to-SFA ratio, *VMR* VFA-to-SMA ratio

### Risk factors for overall survival of PDAC

Univariable Cox regression analysis in the training set identified several factors associated with OS in patients with PDAC, including age, BMI, combined pancreatitis at diagnosis, CA 19-9, CA 12-5, CEA, resection margin, lymphovascular invasion, nerve infiltration, tumor grade, TNM stage, adjuvant therapy, VFA, VFI, SFA, SMD, VSR, and VMR. The multivariable analysis revealed that the following were independently associated with worse OS: high CA 19-9 level (> 538.4 U/L) (hazard ratio (HR)  =  1.483, 95% CI: 1.120–1.964, *p*  =  0.006), high CA 12-5 level (> 14.2 U/L) (HR  =  1.460, 95% CI: 1.095–1.946, *p*  =  0.01), the presence of lymphovascular invasion (HR  =  1.848, 95% CI: 1.413–2.417, *p*  <  0.001), TNM stage ≥ IIB (HR  =  1.551, 95% CI: 1.185–2.028, *p*  =  0.001), and high VSR  (HR  =  1.462, 95% CI: 1.108–1.931, *p*  =  0.007) (Table 4). In contrast, adjuvant therapy (HR = 0.581, 95% CI: 0.436–0.775, *p* < 0.001), high SFA (HR = 0.649, 95% CI: 0.490–0.859, *p* = 0.002), and high SMD (HR = 0.609, 95% CI: 0.432–0.859, *p* = 0.005) were found to be associated with better OS in patients with PDAC.

**Table 4:** Univariable and multivariable Cox regression of factors associated with overall survival in the training set

Characteristics	Univariable analysis		Multivariable analysis	
	HR (95% CI)	*p*-value	HR (95% CI)	*p*-value
Age > 60 years	1.315 (1.031–1.677)	**0.03**	1.005 (0.768–1.316)	0.97
Gender (male)	0.991 (0.775–1.267)	0.94		
BMI > 23.8 kg/m^2^	0.752 (0.569–0.995)	**0.046**	0.928 (0.682–1.263)	0.64
Smoking history	1.017 (0.781–1.325)	0.9		
Diabetes history	1.133 (0.835–1.538)	0.42		
Pancreatitis at diagnosis	1.595 (1.053–2.416)	**0.03**	1.533 (0.995–2.362)	0.05
Tumor Location (body or tail)	0.913 (0.688–1.212)	0.53		
Tumor Size > 2.9 cm	1.266 (0.993–1.614)	0.06		
CA 19-9 > 538.4 U/L	2.142 (1.668–2.751)	**< 0.001**	1.483 (1.120–1.964)	**0.006**
CA 12-5 > 14.2 U/L	1.902 (1.459–2.478)	**< 0.001**	1.460 (1.095–1.946)	**0.01**
CEA > 9.6 ug/L	1.747 (1.246–2.450)	**0.001**	1.129 (0.784–1.627)	0.51
Albumin > 38.7 g/L	0.838 (0.654–1.074)	0.16		
PLR > 193.0	1.271 (0.962–1.679)	0.09		
NLR > 1.6	1.296 (0.930–1.804)	0.13		
LMR > 5.5	0.766 (0.528–1.110)	0.16		
PNI > 42.7	0.828 (0.642–1.068)	0.15		
Resection margin (R1)	1.575 (1.148–2.161)	**0.005**	1.137 (0.815–1.585)	0.45
Lymphovascular invasion	2.459 (1.920–3.149)	**< 0.001**	1.848 (1.413–2.417)	**< 0.001**
Nerve infiltration	2.854 (1.345–6.057)	**0.006**	1.887 (0.876–4.063)	0.11
Tumor grade (Poor)	1.352 (1.041–1.757)	**0.02**	1.225 (0.926–1.620)	0.16
TNM stage (≥ IIB)	1.760 (1.374–2.255)	**< 0.001**	1.551 (1.185–2.028)	**0.001**
Adjuvant therapy	0.614 (0.465–0.811)	**0.001**	0.581 (0.436–0.775)	**< 0.001**
VFA (high)	1.522 (1.151–2.011)	**0.003**	1.772 (0.831–3.780)	0.14
VFI (high)	1.438 (1.088–1.901)	**0.01**	1.094 (0.903–1.143)	0.12
SFA (high)	0.700 (0.546–0.897)	**0.005**	0.649 (0.490–0.859)	**0.002**
SFI (high)	0.795 (0.621–1.018)	0.07		
SMA (high)	0.951 (0.823–1.342)	0.69		
SMI (high)	0.983 (0.860–1.432)	0.42		
IMFA (high)	1.015 (0.778–1.323)	0.91		
IMFI (high)	1.234 (0.967–1.575)	0.09		
SMD (high)	0.629 (0.460–0.861)	**0.004**	0.609 (0.432–0.859)	**0.005**
VSR (high)	1.664 (1.304–2.123)	**< 0.001**	1.462 (1.108–1.931)	**0.007**
VMR (high)	1.518 (1.128–2.042)	**0.006**	1.351 (0.729–2.500)	0.34

Bold was used to highlight values that were statistically significant (*p*-value < 0.05)

*BMI* body mass index, *CA 19-9* carbohydrate antigen 19-9, *CA 12-5* carbohydrate antigen 12-5, *CEA* carcinoembryonic antigen, *LMR* lymphocyte-to-monocyte ratio, *NLR* neutrophil-to-lymphocyte ratio, *PLR* platelet-to-lymphocyte ratio, *PNI* prognostic nutritional index, *R0* negative surgical margin, *R1* positive surgical margin, *IMFA* intermuscular fat area,* IMFI* intermuscular fat index, *SFA* subcutaneous fat area, *SFI* subcutaneous fat index, *SMA* skeletal muscle area, *SMD* skeletal muscle density, *SMI* skeletal muscle index, *TNM* tumor-node-metastasis, *VFA* visceral fat area, *VFI* visceral fat index, *VSR* VFA-to-SFA ratio, *VMR* VFA-to-SMA ratio

**Fig. 3:**
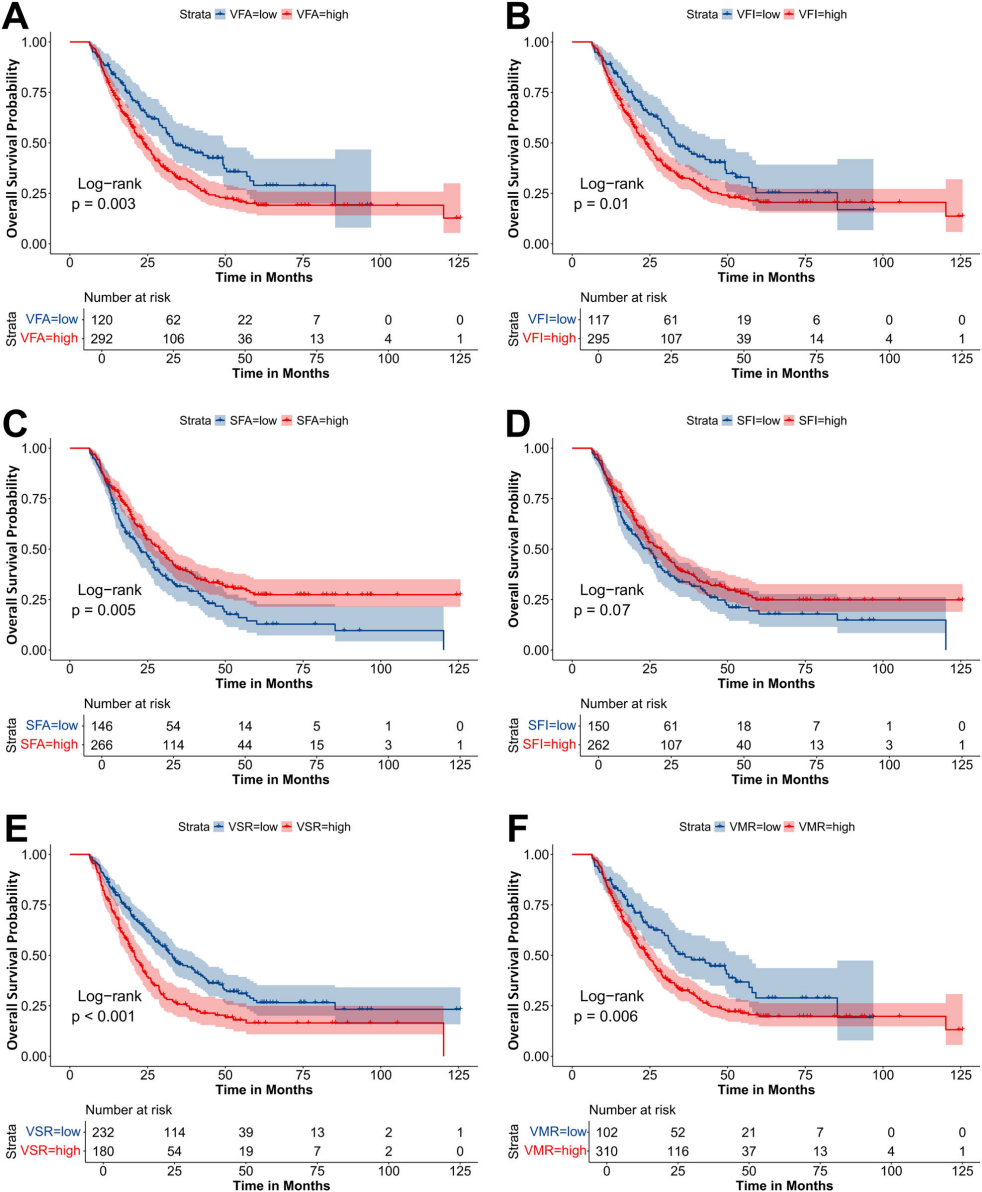
Kaplan–Meier survival curves for the overall survival of patients with PDAC in the training set. The Kaplan–Meier survival curves for overall survival grouped by low and high (**A**) VFA, (**B**) VFI, (**C**) SFA, (**D**) SFI, (**E**) VSR and (**F**) VMR. PDAC, pancreatic ductal adenocarcinoma; VFA, visceral fat area; VFI, visceral fat index; SFA, subcutaneous fat area; SFI, subcutaneous fat index; VSR, VFA-to-SFA ratio; VMR, VFA-to-SMA ratio

Kaplan–Meier survival curves demonstrated that PDAC patients with high VFA, VFI, VSR, and VMR in the training (Fig. 3) and external validation (Fig. S2) sets had worse OS. In contrast, patients in the high SFA and SMD groups had better OS in the training set (Figs. 3 and S3) and external validation set (Figs. S2 and S4) (all *p*  <  0.05).

### Subgroup analyses

**Fig. 4:**
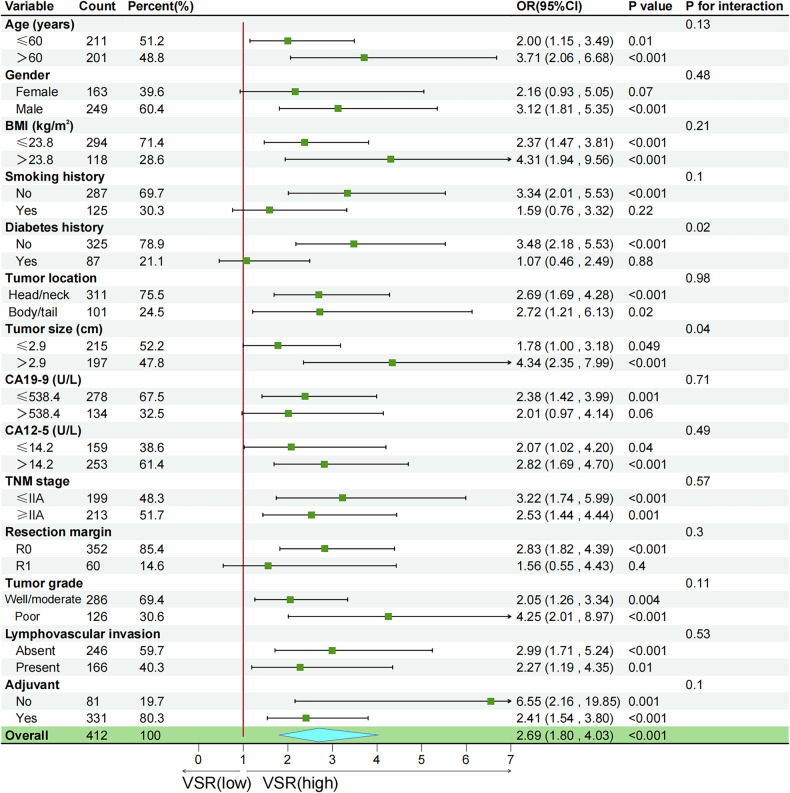
Forest plots showing stratified associations between VSR and early recurrence of PDAC after resection in the training set. BMI, body mass index; CA 19-9, carbohydrate antigen 19-9; CA 12-5, carbohydrate antigen 12-5; PDAC, pancreatic ductal adenocarcinoma; R0, negative surgical margin; R1, positive surgical margin; VSR, VFA-to-SFA ratio; TNM, tumor-node-metastasis; VFA, visceral fat area; SFA, subcutaneous fat area

Subgroup analyses were performed to examine the effect of body composition parameters on ER and OS. As illustrated in Fig. 4, a high VSR demonstrated a strong impact on ER in individuals without diabetes (OR: 3.48 vs. 1.07, *p* for interaction = 0.02). Among PDAC patients with high VSR, a tumor diameter > 2.9 cm further increased the risk of early postoperative recurrence (OR: 4.34 vs. 1.78, *p* for interaction = 0.04) (Fig. 4) and was associated with poorer OS (HR: 2.12 vs. 1.30, *p* for interaction = 0.02) (Fig. S5). No significant differences were observed between SMD or SFA and OS across the subgroups (all interaction *p*-values > 0.05). High SMD and SFA were consistently associated with improved OS across all subgroups (Figs. S6 and S7).

### Body composition and clinical indicator combinations for predicting early recurrence and overall survival

**Fig. 5:**
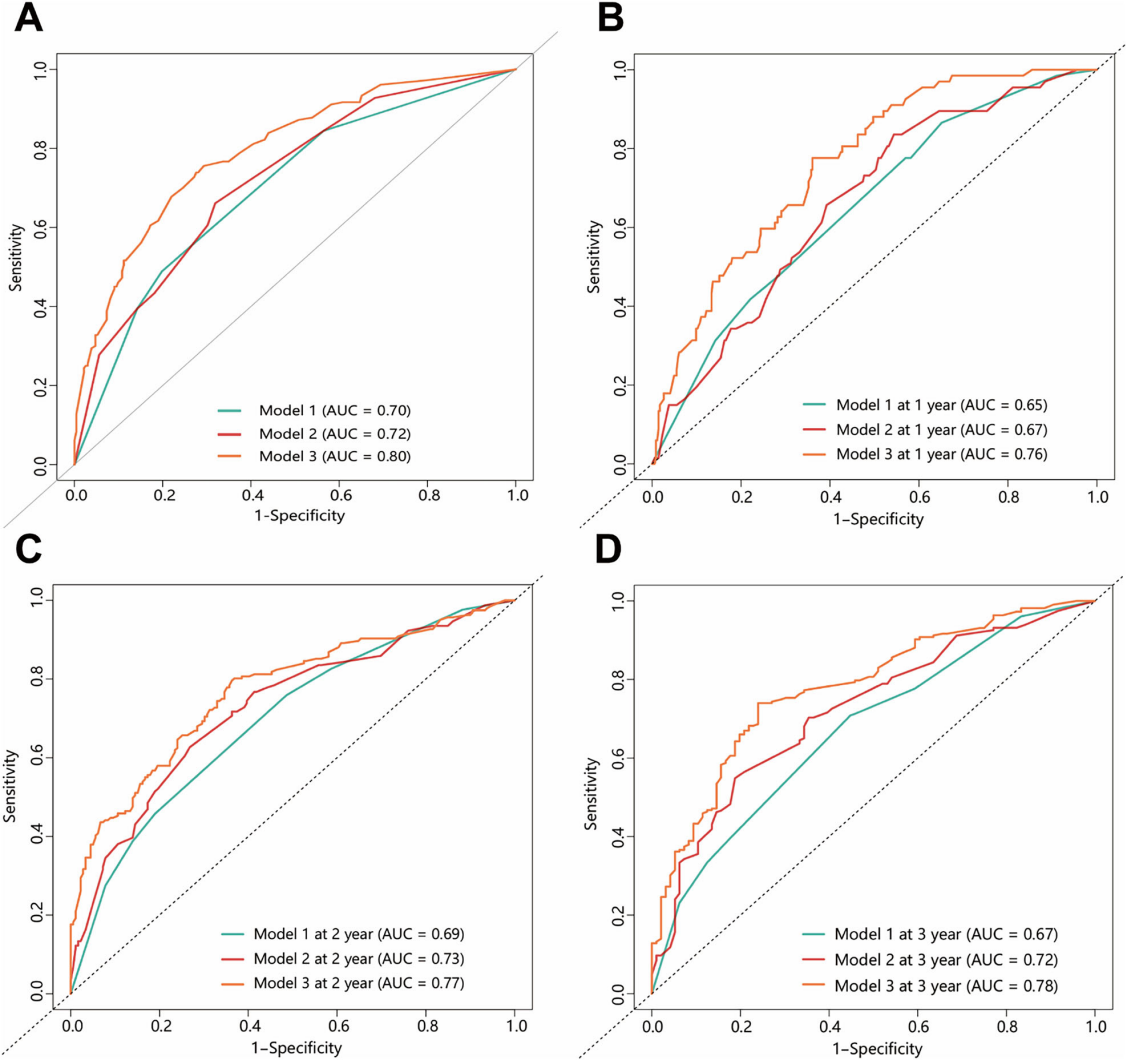
ROC curves of the prediction models for early recurrence and overall survival in the training set. **A** AUCs of the models for predicting early recurrence (Model1: CA 19-9 and CA 12-5; Model 2: CA 19-9 and CA 12-5 and VSR. Model 3: CA 19-9, CA 12-5, VSR, TNM stage, lymphovascular invasion, and adjuvant therapy). **B**–**D** AUCs of the models for predicting overall survival (Model1: CA 19-9 and CA 12-5; Model 2: CA 19-9 and CA 12-5, SFA, SMD, and VSR; Model 3: CA 19-9 and CA 12-5, SFA, SMD, VSR, TNM stage, lymphovascular invasion, and adjuvant therapy). AUC, area under the ROC curve; CA 19-9, carbohydrate antigen 19-9; CA 12-5, carbohydrate antigen 12-5; SFA, subcutaneous fat area; SMD, skeletal muscle density; TNM, tumor-node-metastasis; VSR, VFA-to-SFA; VFA, visceral fat area

**Fig. 6:**
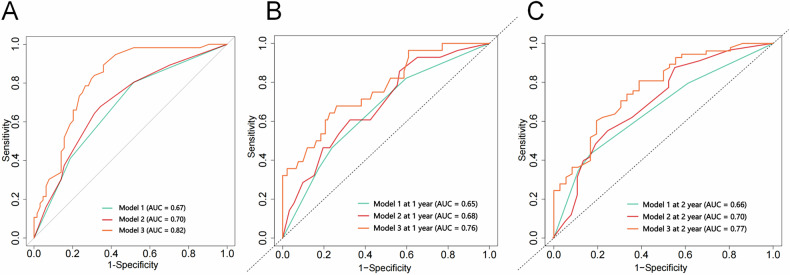
ROC curves of the prediction models for early recurrence and overall survival in the external validation set. **A** AUCs of the models for predicting early recurrence (Model1: CA 19-9 and CA 12-5; Model 2: CA 19-9 and CA 12-5 and VSR. Model 3: CA 19-9, CA 12-5, VSR, TNM stage, lymphovascular invasion, and adjuvant therapy). **B**,** C** AUCs of the models for predicting overall survival (Model1: CA 19-9 and CA 12-5; Model 2: CA 19-9 and CA 12-5, SFA, SMD, and VSR; Model 3: CA 19-9 and CA 12-5, SFA, SMD, VSR, TNM stage, lymphovascular invasion, and adjuvant therapy). AUC, area under the ROC curve; CA 19-9, carbohydrate antigen 19-9; CA 12-5, carbohydrate antigen 12-5; SFA, subcutaneous fat area; SMD, skeletal muscle density; TNM, tumor-node-metastasis; VSR, VFA-to-SFA; VFA, visceral fat area

ROC curve analyses demonstrated the predictive abilities of body composition and clinicopathological indicator combinations for predicting ER and OS in patients with PDAC. For predicting ER, Model 1 (CA 19-9 and CA 12-5) had areas under the curve (AUCs) of 0.70 in the training set and 0.67 in the validation set; Model 2 (CA 19-9, CA 12-5, and VSR) achieved AUCs of 0.72 in the training set and 0.70 in the validation set; and Model 3 (CA 19-9, CA 12-5, VSR, TNM stage, lymphovascular invasion, and adjuvant therapy) showed AUCs of 0.80 in the training set and 0.82 in the validation set (Figs. 5A and 6A).

For OS prediction, in the training set (Fig. 5B–D), Model 1 (CA 19-9 and CA 12-5) achieved AUCs of 0.65 at 1 year, 0.69 at 2 years, and 0.67 at 3 years; Model 2 (CA 19-9, CA 12-5, SFA, SMD, and VSR) had AUCs of 0.67 at 1 year, 0.73 at 2 years, and 0.72 at 3 years; Model 3 (CA 19-9, CA 12-5, SFA, SMD, VSR, TNM stage, lymphovascular invasion, and adjuvant therapy) reached AUCs of 0.76 at 1 year, 0.77 at 2 years, and 0.78 at 3 years. Due to the external validation set’s shorter median follow-up time of 26.4 months (vs. 50.4 months in the training set), only 1- and 2-year OS predictions were feasible (Fig. 6B, C). Model 1 yielded AUCs of 0.65 at 1 year and 0.66 at 2 years; Model 2 demonstrated AUCs of 0.68 at 1 year and 0.70 at 2 years; Model 3 showed AUCs of 0.76 at 1 year and 0.77 at 2 years. In summary, Model 3, which incorporated body composition and clinicopathological indicators, outperformed the other two models in terms of ER and OS prediction across training and validation sets. Calibration curves confirmed good predictive accuracy between the models’ actual and predicted probabilities (Figs. S8 and S9).

## Discussion

This retrospective study, involving a large cohort of patients with PDAC, confirmed the significant prognostic role of body composition in outcomes following pancreatectomy. Specifically, a high VSR was identified as an independent risk factor for early postoperative recurrence and worse survival outcomes. Conversely, high SMD and SFA were associated with better survival. Predictive models for ER and OS were developed by integrating body composition parameters with clinicopathological indicators. These models demonstrated good predictive performance for both ER and OS, offering a reliable tool for identifying patients with PDAC at a high risk of adverse outcomes.

Previous studies have shown that body composition is associated with early postoperative recurrence in cancers such as hepatocellular carcinoma and gallbladder carcinoma [18, 19]. However, investigations into the relationship between body composition and early postoperative recurrence in PDAC are lacking. Therefore, this study, based on a decade-long longitudinal cohort, was conducted to investigate the relationship between body composition, postoperative recurrence, and long-term survival in patients with PDAC.

This study found that VSR is not only an independent risk factor for early postoperative recurrence in patients with pancreatic cancer but is also an important imaging indicator for long-term survival. However, conclusions from previous studies have been inconsistent owing to differences in study design and populations. For instance, while Okumura et al identified VSR as an independent risk factor for poor prognosis in patients with pancreatic cancer [7], another study involving 111 patients found no significant association between VSR and OS [20]. Nevertheless, the present study supports recent findings associating VSR with poor prognosis in various cancers [21–23], specifically between VSR and ER after PDAC surgery.

This association may be related to the differences in physiological functions and tumor regulation between visceral and subcutaneous fat [24]. Visceral fat secretes pro-inflammatory cytokines, such as interleukin-6 and tumor necrosis factor-α, which modulate the tumor microenvironment and promote immune evasion [25, 26]. Additionally, leptin produced by visceral fat synergizes with vascular endothelial growth factor (VEGF) to promote tumor cell proliferation, metastasis, anti-apoptosis, and angiogenesis, thereby accelerating tumor progression [27]. Visceral fat may also lead to insulin resistance and impaired triglyceride storage [28], as evidenced by the higher proportion of patients with diabetes in the high-VSR group (Table 2). In contrast, subcutaneous fat primarily serves as an energy reserve, supporting metabolic demands and facilitating postoperative recovery [29, 30]. Higher subcutaneous fat levels have been associated with improved postoperative outcomes. Therefore, an elevated VSR reflects an imbalance between the visceral and subcutaneous fat, indicating a higher risk of ER and poorer survival outcomes.

Subgroup analyses revealed that in patients with a high VSR, the risk of ER and adverse outcomes in PDAC was further exacerbated when accompanied by larger tumor diameters. This may reflect a synergistic interaction between tumor burden and dysregulated adipose metabolism. Larger tumors increase energy demands, mobilizing visceral fat reserves and elevating levels of free fatty acids (FFAs) and triglycerides, which fuel tumor cells while amplifying oxidative stress and inflammation to promote progression [31]. This interplay may create a vicious cycle, exacerbating tumor growth and adverse outcomes in high-VSR patients [32].

Notably, the effect of a high VSR on ER risk was more pronounced in patients without diabetes. Chronic insulin resistance and inflammation in diabetes may mask VSR’s independent impact on PDAC recurrence [33, 34]. These findings highlight the role of VSR as an independent prognostic marker for pancreatic cancer, providing additional clinical value in specific patient subgroups. VSR’s integration into clinical decision-making could enhance recurrence risk assessment and inform tailored treatment strategies. Further studies are required to elucidate the mechanisms underlying these complex interactions.

This study identified SMD, a key indicator of skeletal muscle quality, as an independent predictor of postoperative survival outcomes in patients with pancreatic cancer. However, SMA was not associated with outcomes, consistent with previous findings in patients with pancreatic cancer [35, 36]. Despite standardizing SMA by height to control for individual differences [37], the standardized SMI showed no significant correlation with prognosis. Kim et al similarly demonstrated that SMD, compared to SMI, more accurately predicts survival in patients with pancreatic cancer [36]. Similar trends have been reported in other cancer studies, where low SMD, rather than a low SMI, correlates with poorer survival [38, 39], as a larger muscle area does not necessarily reflect better muscle quality. Furthermore, SMD decline is detected earlier than SMI decline in cancer patients [38], making SMD a more reliable marker of skeletal muscle health. The prognostic relevance of low SMD may be linked to intramuscular fat infiltration and metabolic disturbances. Low SMD indicates triglyceride accumulation within muscle cells, suggesting increased lipid content in muscle fibers, which can impair muscle function [40, 41]. Although indicators such as IMFA and IMFI assess intramuscular fat, they are based on isolated muscle tissue and fail to capture the heterogeneity of muscle distribution, thus limiting their prognostic value [35]. These findings emphasize that skeletal muscle quality, rather than quantity, is critical for the prognosis of pancreatic cancer. SMD serves as a reliable surrogate for skeletal muscle quality, making it an important marker for the prognostic evaluation of patients with pancreatic cancer.

This study offers important clinical implications for staging, treatment planning, surgical decision-making, and future research. The findings may influence the decision to adopt a “surgery-first” approach or neoadjuvant chemotherapy, particularly in high-risk patients with unfavorable body composition profiles, such as low SMD or high VSR. The reversibility of skeletal muscle deterioration and visceral fat accumulation offers an opportunity for preoperative rehabilitation to enhance patient outcomes and improve survival outcomes. Furthermore, the developed model is useful for prognostic prediction in patients with pancreatic cancer, aiding in more accurate risk stratification and personalized treatment strategies.

Despite its strengths, the study has several limitations. First, as this is a retrospective study, it is susceptible to patient selection bias. Second, while subgroup analyses explored associations within specific patient populations, modest sample sizes in certain subgroups may limit the generalizability, thus warranting future multicenter studies with larger cohorts. Moving forward, prospective validation across diverse clinical settings is essential to solidify these findings and facilitate clinical translation.

## Conclusion

This study highlighted the utility and prognostic value of preoperative CT-based body composition in predicting ER and overall survival in patients with PDAC. The integrated model combining body composition parameters with clinicopathological indicators, including CA 19-9, CA 12-5, TNM stage, lymphovascular invasion, and adjuvant therapy, demonstrated good predictive performance for patient prognosis. These findings could help identify patients at a high risk of ER and assist clinicians in delivering personalized management and treatment strategies.

## Supplementary information

ELECTRONIC SUPPLEMENTARY MATERIAL

## Abbreviations

CA 12-5: Carbohydrate antigen 12-5
CA 19-9: Carbohydrate antigen 19-9
CEA: Carcinoembryonic antigen
ER: Early recurrence
IMFA: Intermuscular fat area
PDAC: Pancreatic ductal adenocarcinoma
PNI: Prognostic nutrition index
SFA: Subcutaneous fat area
SMD: Skeletal muscle density
SMI: Skeletal muscle index
TNM: Tumor-node-metastasis
VFA: Visceral fat area
VFI: Visceral fat index
VMR: Visceral-to-muscle ratio
VSR: Visceral-to-subcutaneous fat tissue area ratio
