# Supplementary material for: Prognostic value of body composition on early recurrence and long-term survival of resectable pancreatic ductal adenocarcinoma
Source: Eur Radiol. 2025 Nov 10;36(4):2945–64. doi: 10.1007/s00330-025-12028-8 (PMC13035614; doi:10.1007/s00330-025-12028-8)
Supplement: Supplementary file 1 — ELECTRONIC SUPPLEMENTARY MATERIAL [file 330_2025_12028_MOESM1_ESM.pdf]

Prognostic value of body composition on early recurrence and long-term survival of resectable pancreatic ductal adenocarcinoma

ELECTRONIC SUPPLEMENTARY MATERIAL

1. Supplementary Methods. - *Pages 2*.
2. Supplementary Table S1. Comparison of the baseline clinicopathological characteristics between the training and external validation sets - *Pages 2-4*.
3. Supplementary Table S2. The cut-off value of body composition based on gender - *Pages 5*.
4. Supplementary Table S3. Inter-observer agreement analysis for original body composition measurements - *Pages 5*.
5. Supplementary Figure S1. Bland-Altman plot for agreement between two observers for five original body composition parameters. - *Pages 6*.
6. Supplementary Figure S2. Kaplan-Meier survival curves for overall survival of patients with PDAC in the external validation set. The Kaplan-Meier survival curves for overall survival grouped by low and high (A) VFA, (B) VFI, (C) SFA, (D) SFI, (E) VSR and (F) VMR - *Pages 7*.
7. Supplementary Figure S3. Kaplan-Meier survival curves for overall survival of patients with PDAC in the training set. The Kaplan-Meier survival curves for overall survival grouped by low and high (A) SMA, (B) SMI, (C) IMFA, (D) IMFI, and (E) SMD - *Pages 8*.
8. Supplementary Figure S4. Kaplan-Meier survival curves for overall survival of patients with PDAC in the external validation set. The Kaplan-Meier survival curves for overall survival grouped by low and high (A) SMA, (B) SMI, (C) IMFA, (D) IMFI, and (E) SMD - *Pages 9*.
9. Supplementary Figure S5. Forest plots showing stratified associations between VSR and overall survival of PDAC in the training set. - *Pages 10*.
10. Supplementary Figure S6. Forest plots showing stratified associations between SMD and overall survival of PDAC in the training set. - *Pages 11*.
11. Supplementary Figure S7. Forest plots showing stratified associations between SFA and overall survival of PDAC in the training set. - *Pages 12*.
12. Supplementary Figure S8. Calibration curves of the prediction models for early recurrence and overall survival in the training set. - *Pages 13*.
13. Supplementary Figure S9. Calibration curves of the prediction models for early recurrence and overall survival in the external validation set. - *Pages 14*.

Supplementary methods

Definition of resectable PDAC

Resectable disease was defined as tumors with no major arterial contact and no contact or less than or equal to 180° contact with the portal vein or superior mesenteric vein without vein contour irregularity, according to the National Comprehensive Cancer Network criteria (9). Patients with PDAC were screened for eligibility, initially based on radiologic and medical records and then through confirmation with a retrospective review of CT scans obtained by two experienced radiologist (P.H., with 35 years of experience in pancreatic imaging and C.Z with 30 years of experience in abdominal imaging) and an experienced surgeon (H.W., with 30 years of experience in pancreatic surgery).

Table S1. Comparison of the baseline clinicopathological characteristics between the training and external validation sets

Characteristics	**Total** (n = 532)	**Training set** (n = 412)	**External validation set** (n = 120)	***P*-value**
Age (years)				0.28
≤ 60	265 (49.81)	211 (51.21)	54 (45.0)	
> 60	267 (50.19)	201 (48.79)	66 (55.0)	
Gender				0.76
Female	213 (40.04)	163 (39.56)	50 (41.67)	
Male	319 (59.96)	249 (60.44)	70 (58.33)	
BMI (kg/m^2)				0.5
≤ 23.8	384 (72.18)	294 (71.36)	90 (75.0)	
> 23.8	148 (27.82)	118 (28.64)	30 (25.0)	
Smoking history				0.63
No	374 (70.3)	287 (69.66)	87 (72.5)	
Yes	158 (29.7)	125 (30.34)	33 (27.5)	
Diabetes history				0.33
No	414 (77.82)	325 (78.88)	89 (74.17)	
Yes	118 (22.18)	87 (21.12)	31 (25.83)	
Pancreatitis at diagnosis				0.59
No	479 (90.04)	373 (90.53)	106 (88.33)	
Yes	53 (9.96)	39 (9.47)	14 (11.67)	
Tumor Location				0.89
Head or neck	403 (75.75)	311 (75.49)	92 (76.67)	
Body or tail	129 (24.25)	101 (24.51)	28 (23.33)	
Tumor Size (cm)				0.34
≤ 2.9	271 (50.94)	215 (52.18)	56 (46.67)	
> 2.9	261 (49.06)	197 (47.82)	64 (53.33)	

CA19-9(U/ml)				0.56
≤ 538.4	363 (68.23)	278 (67.48)	85 (70.83)	
> 538.4	169 (31.77)	134 (32.52)	35 (29.17)	
CA 12-5 (U/ml)				0.51
≤ 14.2	210 (39.47)	159 (38.59)	51 (42.5)	
> 14.2	322 (60.53)	253 (61.41)	69 (57.5)	
CEA (ug/L)				0.29
≤ 9.6	471 (88.53)	361 (87.62)	110 (91.67)	
> 9.6	61 (11.47)	51 (12.38)	10 (8.33)	
Albumin (g/L)				0.22
≤ 38.7	312 (58.65)	248 (60.19)	64 (53.33)	
> 38.7	220 (41.35)	164 (39.81)	56 (46.67)	
PLR				0.24
≤ 193.0	388 (72.93)	306 (74.27)	82 (68.33)	
> 193.0	144 (27.07)	106 (25.73)	38 (31.67)	
NLR				0.48
≤ 1.6	97 (18.23)	72 (17.48)	25 (20.83)	
> 1.6	435 (81.77)	340 (82.52)	95 (79.17)	
LMR				0.55
≤ 5.5	463 (87.03)	361 (87.62)	102 (85.0)	
> 5.5	69 (12.97)	51 (12.38)	18 (15.0)	
PNI				0.53
≤ 42.7	188 (35.34)	149 (36.17)	39 (32.5)	
> 42.7	344 (64.66)	263 (63.83)	81 (67.5)	
Margin,				0.85
R0	456 (85.71)	352 (85.44)	104 (86.67)	
R1	76 (14.29)	60 (14.56)	16 (13.33)	
Lymphovascular invasion				0.62
Absent	314 (59.02)	246 (59.71)	68 (56.67)	
Present	218 (40.98)	166 (40.29)	52 (43.33)	
Nerve infiltration				0.44
Absent	30 (5.64)	21 (5.1)	9 (7.5)	
Present	502 (94.36)	391 (94.9)	111 (92.5)	
Grade				0.65
Well or moderate	366 (68.8)	286 (69.42)	80 (66.67)	
Poor	166 (31.2)	126 (30.58)	40 (33.33)	
T stage				0.5
T1	104 (19.55)	84 (20.39)	20 (16.67)	
T2	313 (58.83)	237 (57.52)	76 (63.33)	
T3	115 (21.62)	91 (22.09)	24 (20.0)	
N stage				0.88
N0	251 (47.18)	195 (47.33)	56 (46.67)	
N1	213 (40.04)	163 (39.56)	50 (41.67)	
N2	68 (12.78)	54 (13.11)	14 (11.67)	
TNM				0.83
≤ IIA	255 (47.93)	199 (48.3)	56 (46.67)	
≥ IIB	277 (52.07)	213 (51.7)	64 (53.33)	
Adjuvant				0.18
No	112 (21.05)	81 (19.66)	31 (25.83)	

Yes	420 (78.95)	331 (80.34)	89 (74.17)	
VFA (cm^2)				0.81
low	157 (29.51)	120 (29.13)	37 (30.83)	
high	375 (70.49)	292 (70.87)	83 (69.17)	
VFI (cm^2/m^2)				0.45
low	156 (29.32)	117 (28.4)	39 (32.5)	
high	376 (70.68)	295 (71.6)	81 (67.5)	
SFA (cm^2)				0.14
low	198 (37.22)	146 (35.44)	52 (43.33)	
high	334 (62.78)	266 (64.56)	68 (56.67)	
SFI (cm^2/m^2)				0.44
low	199 (37.41)	150 (36.41)	49 (40.83)	
high	333 (62.59)	262 (63.59)	71 (59.17)	
SMA (cm^2)				0.43
low	318 (59.77)	242 (58.74)	76 (63.33)	
high	214 (40.23)	170 (41.26)	44 (36.67)	
SMI (cm^2/m^2)				0.82
low	357 (67.11)	278 (67.48)	79 (65.83)	
high	175 (32.89)	134 (32.52)	41 (34.17)	
IMFA (cm^2)				0.59
low	160 (30.08)	121 (29.37)	39 (32.5)	
high	372 (69.92)	291 (70.63)	81 (67.5)	
IMFI (cm^2/m^2)				0.48
low	302 (56.77)	230 (55.83)	72 (60.0)	
high	230 (43.23)	182 (44.17)	48 (40.0)	
SMD (HU)				0.3
low	92 (17.29)	67 (16.26)	25 (20.83)	
high	440 (82.71)	345 (83.74)	95 (79.17)	
VSR				0.26
low	292 (54.89)	232 (56.31)	60 (50.0)	
high	240 (45.11)	180 (43.69)	60 (50.0)	
VMR				0.5
low	136 (25.56)	102 (24.76)	34 (28.33)	
high	396 (74.44)	310 (75.24)	86 (71.67)	
Early recurrence				0.64
No	296 (55.64)	232 (56.31)	64 (53.33)	
Yes	236 (44.36)	180 (43.69)	56 (46.67)	
Median follow-up time	36.7 (34.1, 45.9)	50.7 (44.7, 54.8)	26.4 (23.6, 27.9)	**<0.001**

Data are presented as n (%) or median with IQR.
Bold was used to highlight values that were statistically significant (*P* value < 0.05). BMI: body mass index; CA 19-9: carbohydrate antigen 19-9; CA 12-5: carbohydrate antigen 12-5; CEA: carcinoembryonic antigen; LMR: lymphocyte-to-monocyte ratio; NLR: neutrophil-to-lymphocyte ratio; PLR: platelet-to-lymphocyte ratio; PNI: prognostic nutritional index; R0 negative surgical margin, R1 positive surgical margin; IMFA, intermuscular fat area; IMFI, intermuscular fat index; SFA, subcutaneous fat area; SFI, subcutaneous fat index; SMA, skeletal muscle area; SMD, skeletal muscle density; SMI, skeletal muscle index; VFA, visceral fat area; VFI, visceral fat index; VSR: VFA-to-SFA ratio; VMR: VFA-to-SMA ratio.

Table S2. The cut-off value of body composition based on gender

	Male	Female
VFA (cm^2)	84.06	41.35
VFI (cm^2/m^2)	28.85	14.17
SFA (cm^2)	57.86	145.55
SFI (cm^2/m^2)	18.89	59.10
SMA (cm^2)	159.00	78.90
SMI (cm^2/m^2)	49.38	38.54
IMFA (cm^2)	3.03	5.73
IMFI (cm^2/m^2)	2.49	2.30
SMD (HU)	32.68	26.05
VSR	0.97	0.95
VMR	0.57	0.45

IMFA, intermuscular fat area; IMFI, intermuscular fat index; SFA, subcutaneous fat area; SFI, subcutaneous fat index; SMA, skeletal muscle area; SMD, skeletal muscle density; SMI, skeletal muscle index; VFA, visceral fat area; VFI, visceral fat index; VSR: VFA-to-SFA ratio; VMR: VFA-to-SMA ratio.

Table S3. Inter-observer agreement analysis for original body composition measurements

	Observer1	Observer2	ICC (95%CI)	Mean Difference(cm^2)	Lower-upper 95% LoA(cm^2)
VFA (cm^2)	106.27±63.06	105.69±63.07	0.999 (0.998-0.999)	0.58	-3.05-4.21
SFA (cm^2)	114.14±55.52	114.49±55.58	0.998 (0.997-0.999)	-0.35	-3.56-2.86
SMA (cm^2)	118.16±26.60	117.82±26.64	0.997 (0.996-0.998)	0.34	-2.76-3.43
IMFA (cm^2)	7.02±4.59	7.10±4.61	0.983 (0.979-0.986)	-0.08	-1.74-1.58
SMD (HU)	35.10±6.71	35.16±6.71	0.999 (0.998-0.999)	-0.06	-0.63-0.52

IMFA, intermuscular fat area; SFA, subcutaneous fat area; SMA, skeletal muscle area; SMD, skeletal muscle density; VFA, visceral fat area; LoA: limits of agreement.

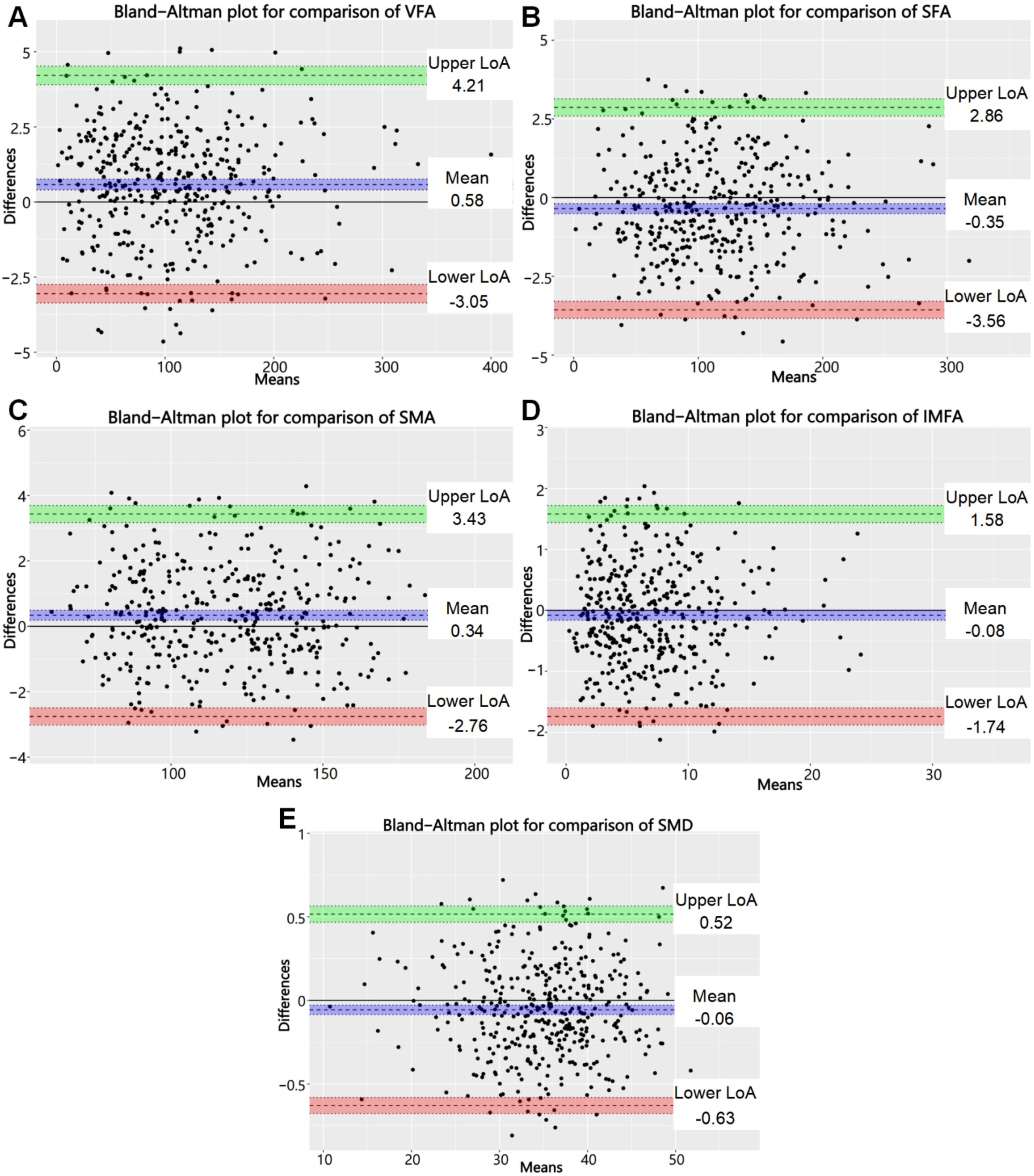

Figure S1. Bland-Altman plot for agreement between two observers for five original body composition parameters: VFA (A), SFA (B), SMA (C), IMFA (D), and SMD (E) in patients with PDAC (n = 412). The middle purple region represents the mean bias. The green and pink regions represent upper and lower limits of agreement (LoA) between two observers. *IMFA, intermuscular fat area; SFA, subcutaneous fat area; SMA, skeletal muscle area; SMD, skeletal muscle density; VFA, visceral fat area.*

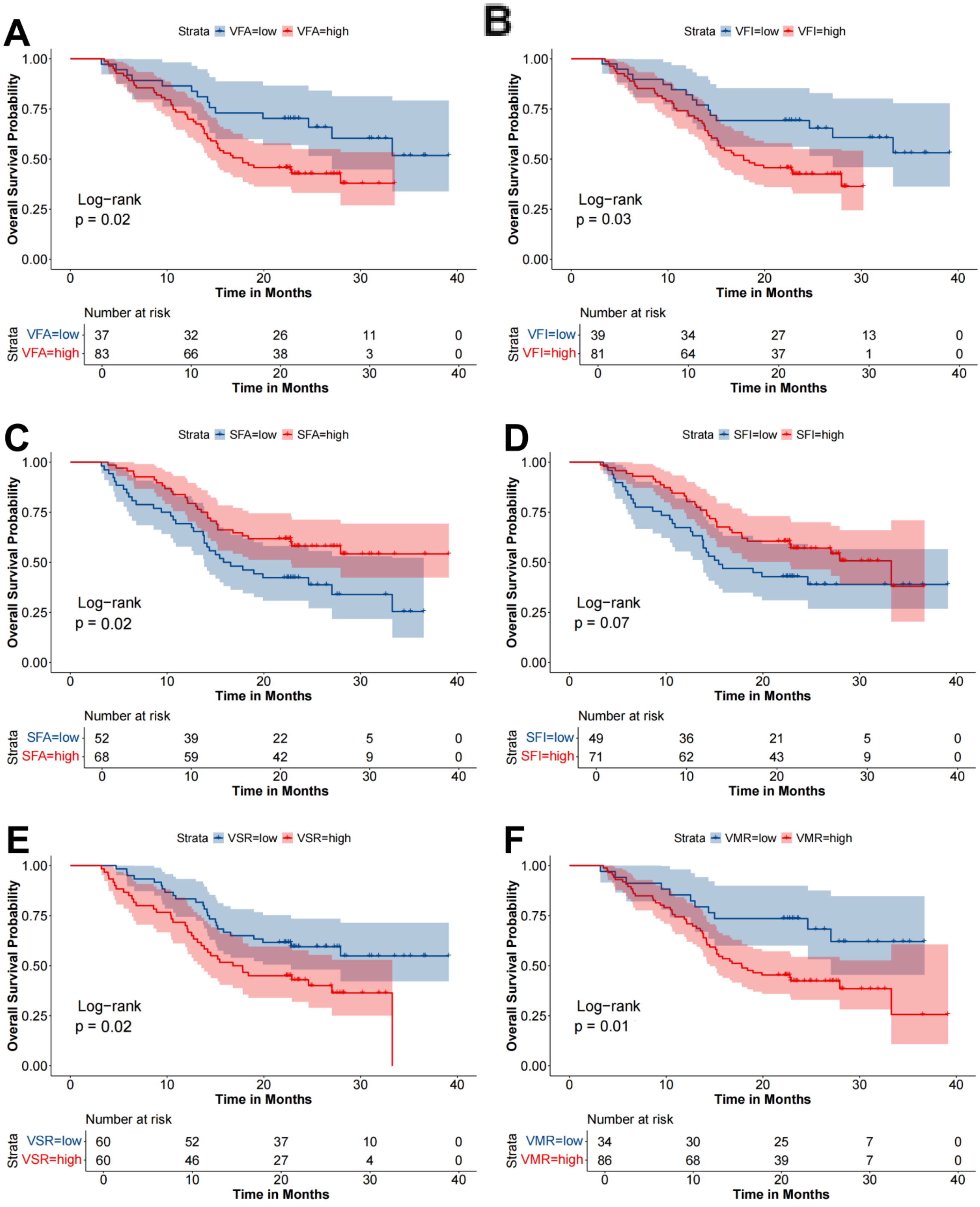

Figure S2. Kaplan-Meier survival curves for overall survival of patients with PDAC in the external validation set. The Kaplan-Meier survival curves for overall survival grouped by low and high (A) VFA, (B) VFI, (C) SFA, (D) SFI, (E) VSR and (F) VMR. *PDAC: pancreatic ductal adenocarcinoma; VFA, visceral fat area; VFI, visceral fat index; SFA, subcutaneous fat area; SFI, subcutaneous fat index; VSR: VFA-to-SFA ratio; VMR: VFA-to-SMA ratio.*

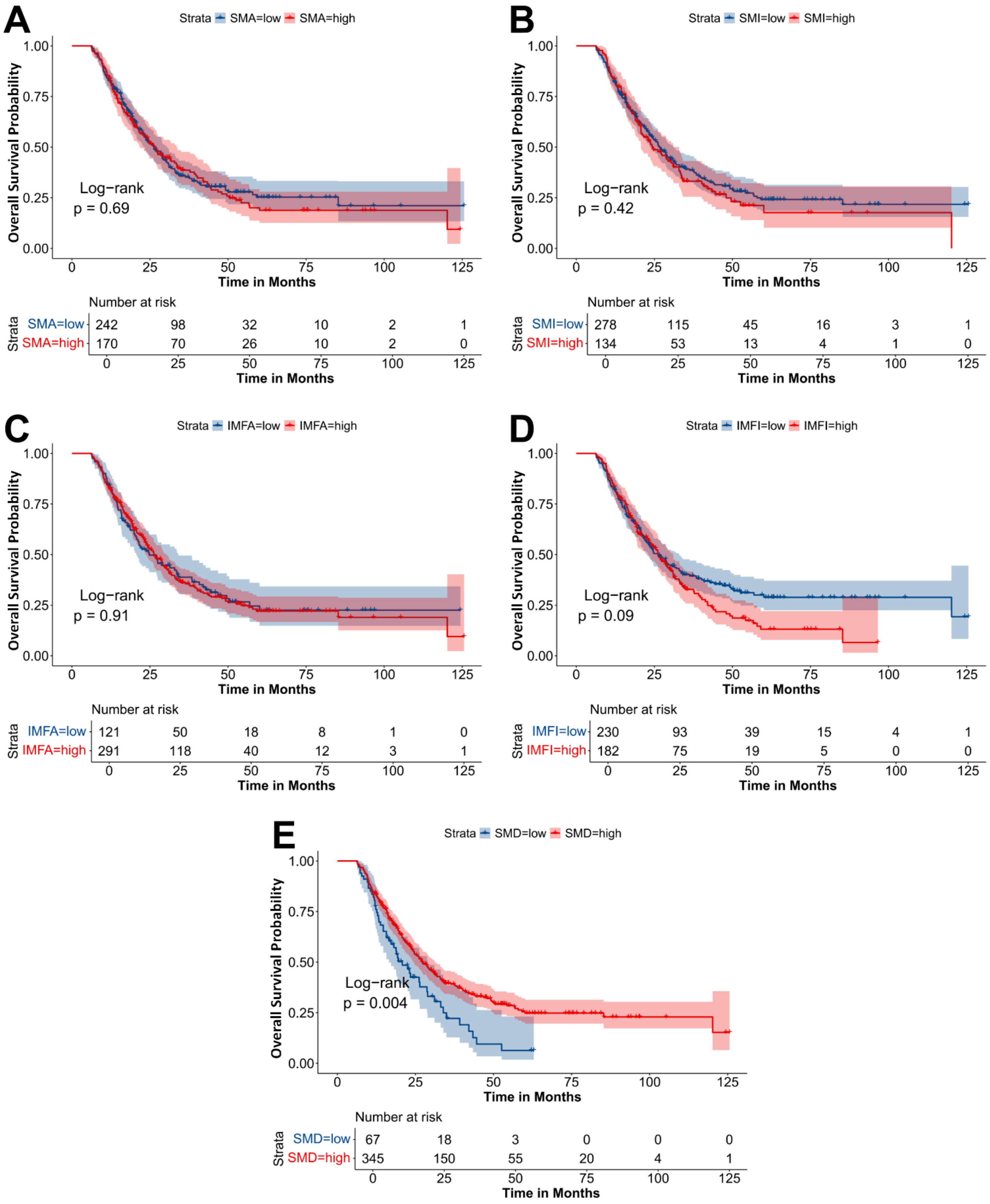

Figure S3. Kaplan-Meier survival curves for overall survival of patients with PDAC in the training set. The Kaplan-Meier survival curves for overall survival grouped by low and high (A) SMA, (B) SMI, (C) IMFA, (D) IMFI, and (E) SMD. *IMFA, intermuscular fat area, IMFI, intermuscular fat index, PDAC: pancreatic ductal adenocarcinoma; SMA, skeletal muscle area, SMD, skeletal muscle density, SMI, skeletal muscle index.*

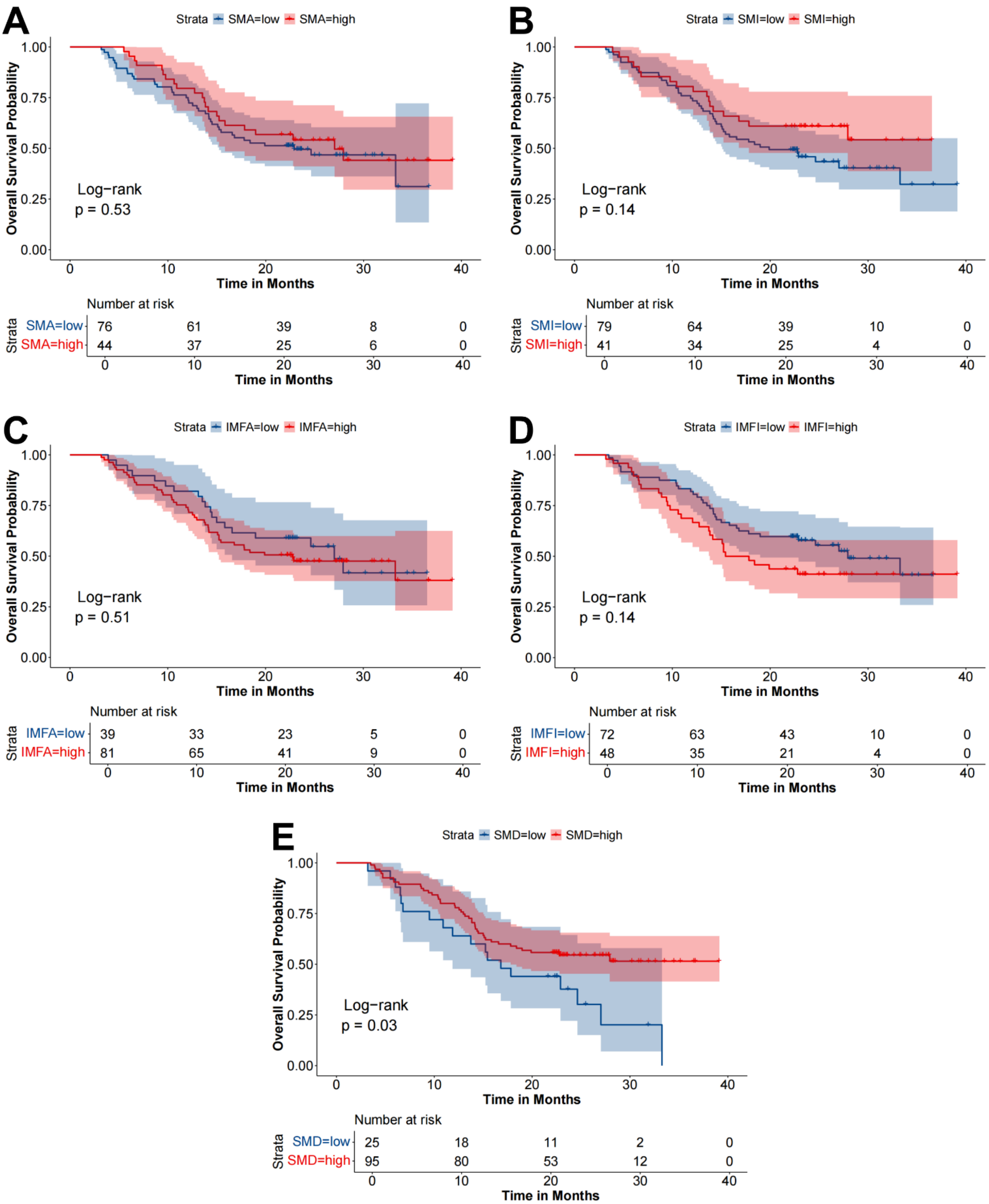

Figure S4. Kaplan-Meier survival curves for overall survival of patients with PDAC in the external validation set. The Kaplan-Meier survival curves for overall survival grouped by low and high (A) SMA, (B) SMI, (C) IMFA, (D) IMFI, and (E) SMD. *IMFA, intermuscular fat area, IMFI, intermuscular fat index, PDAC: pancreatic ductal adenocarcinoma; SMA, skeletal muscle area, SMD, skeletal muscle density, SMI, skeletal muscle index.*

Variable	Count	Percent(%)	HR(95%CI)	P value	P for interaction
**Age (years)**					0.95
≤60	211	51.2	1.66 (1.17 , 2.35)	0.005	
>60	201	48.8	1.65 (1.17 , 2.31)	0.004	
**Gender**					0.83
Female	163	39.6	1.96 (1.24 , 3.10)	0.004	
Male	249	60.4	1.83 (1.30 , 2.58)	0.001	
**BMI (kg/m^2)**					0.49
≤23.8	294	71.4	1.68 (1.26 , 2.23)	<0.001	
>23.8	118	28.6	2.00 (1.20 , 3.33)	0.008	
**Smoking history**					0.95
No	287	69.7	1.73 (1.28 , 2.33)	<0.001	
Yes	125	30.3	1.75 (1.07 , 2.85)	0.03	
**Diabetes history**					0.23
No	325	78.9	1.77 (1.35 , 2.33)	<0.001	
Yes	87	21.1	1.24 (0.71 , 2.14)	0.45	
**Tumor location**					0.27
Head/neck	311	75.5	1.82 (1.37 , 2.40)	<0.001	
Body/tail	101	24.5	1.28 (0.78 , 2.11)	0.33	
**Tumor size (cm)**					0.02
≤2.9	215	52.2	1.30 (0.92 , 1.84)	0.14	
>2.9	197	47.8	2.12 (1.50 , 3.00)	<0.001	
**CA19-9 (U/L)**					0.41
≤538.4	278	67.5	1.41 (1.02 , 1.93)	0.04	
>538.4	134	32.5	1.71 (1.14 , 2.55)	0.01	
**CA12-5 (U/L)**					0.75
≤14.2	159	38.6	1.56 (1.00 , 2.46)	0.052	
>14.2	253	61.4	1.57 (1.17 , 2.11)	0.002	
**TNM stage**					0.56
≤IIA	199	48.3	1.60 (1.10 , 2.34)	0.01	
≥IIA	213	51.7	1.78 (1.29 , 2.46)	<0.001	
**Resection margin**					0.78
R0	352	85.4	1.61 (1.23 , 2.10)	0.001	
R1	60	14.6	1.74 (0.95 , 3.19)	0.07	
**Tumor grade**					0.32
Well/moderate	286	69.4	1.52 (1.13 , 2.04)	0.006	
Poor	126	30.6	1.78 (1.14 , 2.77)	0.01	
**Lymphovascular invasion**					0.76
Absent	246	59.7	1.68 (1.19 , 2.36)	0.003	
Present	166	40.3	1.50 (1.06 , 2.13)	0.02	
**Adjuvant**					0.18
No	81	19.7	2.04 (1.24 , 3.36)	0.005	
Yes	331	80.3	1.61 (1.22 , 2.14)	0.001	
**Overall**	412	100	1.66 (1.30 , 2.12)	<0.001	

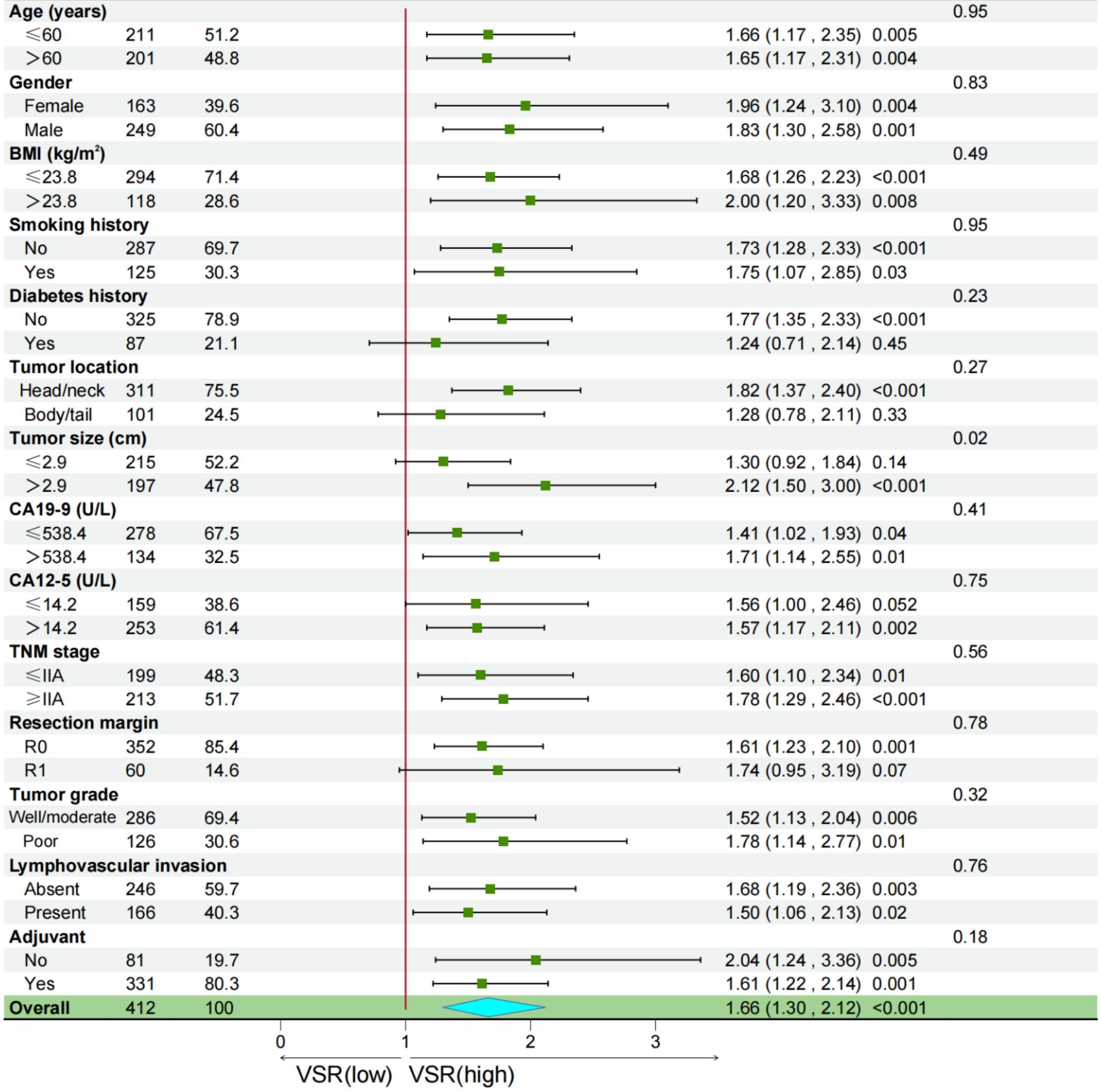

Figure S5. Forest plots showing stratified associations between VSR and overall survival of PDAC in the training set. *BMI: body mass index; CA19-9: carbohydrate antigen 19-9; CA12-5: carbohydrate antigen 12-5; PDAC: pancreatic ductal adenocarcinoma; R0: negative surgical margin, R1: positive surgical margin; VSR: VFA-to-SFA ratio; TNM: tumor-node-metastasis.*

Variable	Count	Percent(%)	HR(95%CI)	P value	P for interaction
**Age (years)**					0.72
≤60	211	51.2	0.74 (0.41 , 1.31)	0.3	
>60	201	48.8	0.63 (0.43 , 0.93)	0.02	
**Gender**					0.77
Female	163	39.6	0.63 (0.40 , 1.00)	0.05	
Male	249	60.4	0.61 (0.40 , 0.94)	0.03	
**BMI (kg/m²)**					0.83
≤23.8	294	71.4	0.60 (0.41 , 0.89)	0.01	
>23.8	118	28.6	0.57 (0.33 , 0.98)	0.04	
**Smoking history**					0.75
No	287	69.7	0.61 (0.43 , 0.87)	0.006	
Yes	125	30.3	0.68 (0.34 , 1.37)	0.28	
**Diabetes history**					0.36
No	325	78.9	0.59 (0.41 , 0.84)	0.004	
Yes	87	21.1	0.78 (0.42 , 1.47)	0.45	
**Tumor location**					0.3
Head/neck	311	75.5	0.57 (0.40 , 0.81)	0.002	
Body/tail	101	24.5	0.88 (0.44 , 1.73)	0.7	
**Tumor size (cm)**					0.76
≤2.9	215	52.2	0.64 (0.41 , 1.02)	0.06	
>2.9	197	47.8	0.63 (0.41 , 0.97)	0.04	
**CA19-9 (U/L)**					0.47
≤538.4	278	67.5	0.58 (0.39 , 0.88)	0.009	
>538.4	134	32.5	0.74 (0.45 , 1.21)	0.23	
**CA12-5 (U/L)**					0.08
≤14.2	159	38.6	0.97 (0.50 , 1.89)	0.93	
>14.2	253	61.4	0.55 (0.38 , 0.79)	0.001	
**TNM stage**					0.07
≤IIA	199	48.3	0.74 (0.46 , 1.20)	0.22	
≥IIA	213	51.7	0.62 (0.38 , 0.84)	<0.001	
**Resection margin**					0.78
R0	352	85.4	0.63 (0.45 , 0.88)	0.008	
R1	60	14.6	0.60 (0.28 , 1.30)	0.19	
**Tumor grade**					0.51
Well/moderate	286	69.4	0.57 (0.39 , 0.84)	0.004	
Poor	126	30.6	0.86 (0.49 , 1.51)	0.6	
**Lymphovascular invasion**					0.51
Absent	246	59.7	0.57 (0.36 , 0.89)	0.01	
Present	166	40.3	0.73 (0.47 , 1.13)	0.16	
**Adjuvant**					0.56
No	81	19.7	0.56 (0.30 , 1.03)	0.06	
Yes	331	80.3	0.65 (0.45 , 0.95)	0.02	
**Overall**	412	100	0.63 (0.46 , 0.86)	0.004	

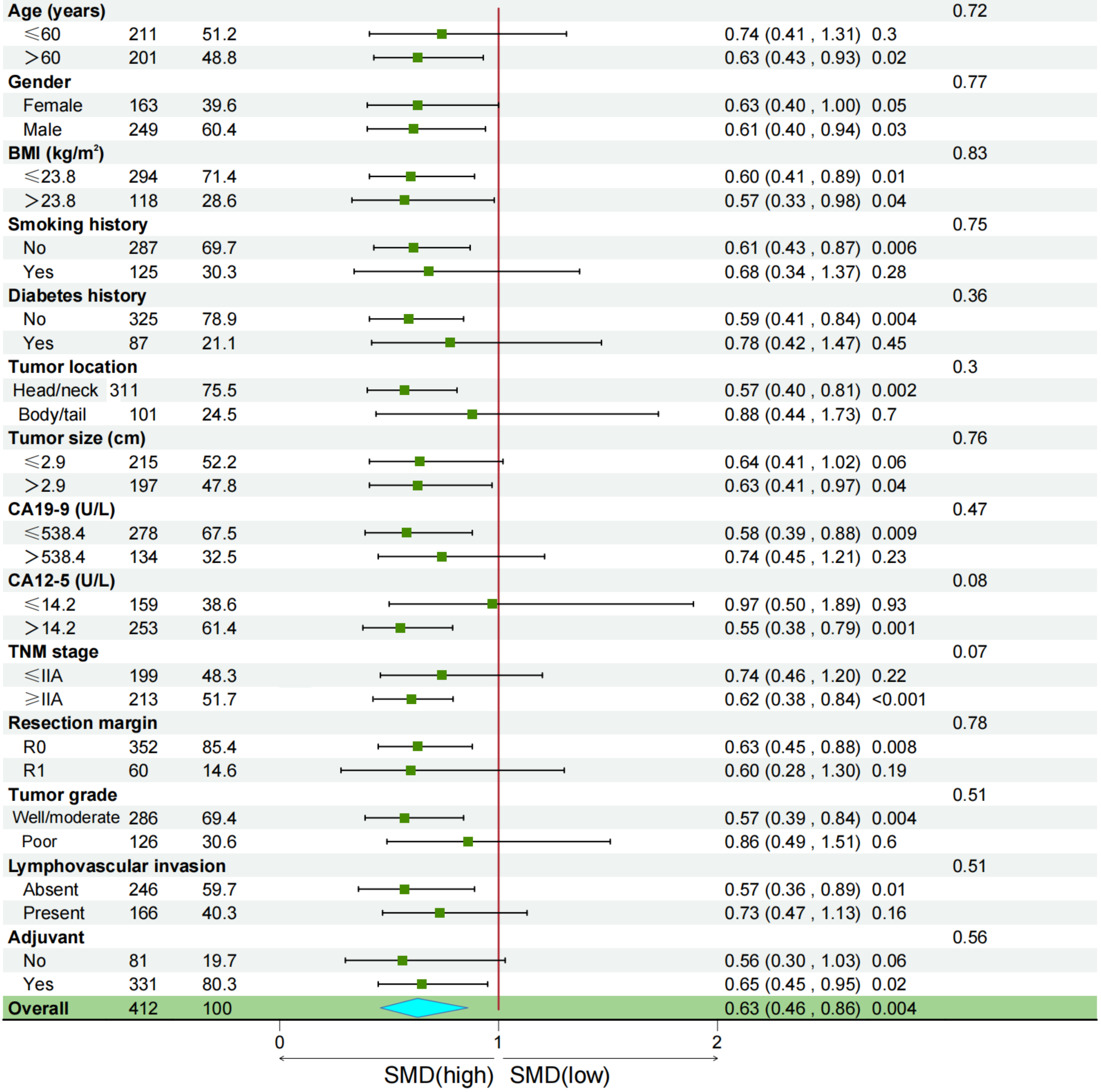

Figure S6. Forest plots showing stratified associations between SMD and overall survival of PDAC in the training set. *BMI: body mass index; CA19-9: carbohydrate antigen 19-9; CA12-5: carbohydrate antigen 12-5; PDAC: pancreatic ductal adenocarcinoma; PDAC: pancreatic ductal adenocarcinoma; R0: negative surgical margin, R1: positive surgical margin; TNM: tumor-node-metastasis. SMD：skeletal muscle density.*

Variable	Count	Percent(%)	HR(95%CI)	P value	P for interaction
**Age (years)**					0.89
⩽60	211	51.2	0.72 (0.50 , 1.02)	0.07	
>60	201	48.8	0.69 (0.49 , 0.98)	0.04	
**Gender**					0.73
Female	163	39.6	0.64 (0.43 , 0.94)	0.02	
Male	249	60.4	0.70 (0.49 , 1.02)	0.06	
**BMI (kg/m²)**					0.8
⩽23.8	294	71.4	0.75 (0.57 , 1.00)	0.047	
>23.8	118	28.6	0.72 (0.33 , 1.57)	0.41	
**Smoking history**					0.27
No	287	69.7	0.64 (0.48 , 0.86)	0.003	
Yes	125	30.3	0.87 (0.54 , 1.42)	0.59	
**Diabetes history**					0.31
No	325	78.9	0.74 (0.56 , 0.97)	0.03	
Yes	87	21.1	0.52 (0.29 , 0.92)	0.02	
**Tumor location**					0.68
No	311	75.5	0.72 (0.54 , 0.96)	0.03	
Yes	101	24.5	0.65 (0.39 , 1.07)	0.09	
**Tmor size (cm)**					0.14
⩽2.9	215	52.2	0.57 (0.40 , 0.81)	0.002	
>2.9	197	47.8	0.85 (0.60 , 1.21)	0.38	
**CA19-9 (U/L)**					0.26
⩽538.4	278	67.5	0.68 (0.49 , 0.94)	0.02	
>538.4	134	32.5	0.91 (0.62 , 1.35)	0.65	
**CA12-5 (U/L)**					0.2
⩽14.2	159	38.6	0.87 (0.55 , 1.38)	0.56	
>14.2	253	61.4	0.63 (0.47 , 0.85)	0.002	
**TNM stage**					0.49
⩽IIA	199	48.3	0.65 (0.44 , 0.96)	0.03	
⩾IIA	213	51.7	0.79 (0.57 , 1.09)	0.15	
**Resection margin**					0.45
R0	352	85.4	0.69 (0.52 , 0.91)	0.008	
R1	60	14.6	0.86 (0.48 , 1.53)	0.61	
**Tumor grade**					0.8
Well/moderate	286	69.4	0.66 (0.49 , 0.88)	0.006	
Poor	126	30.6	0.75 (0.47 , 1.20)	0.23	
**Lymphovascular invasion**					0.35
No	246	59.7	0.68 (0.48 , 0.98)	0.04	
Yes	166	40.3	0.87 (0.61 , 1.23)	0.42	
**Adjuvant**					0.67
No	81	19.7	0.67 (0.41 , 1.10)	0.11	
Yes	331	80.3	0.70 (0.53 , 0.93)	0.02	
**Overall**	412	100	0.70 (0.55 , 0.90)	0.005	

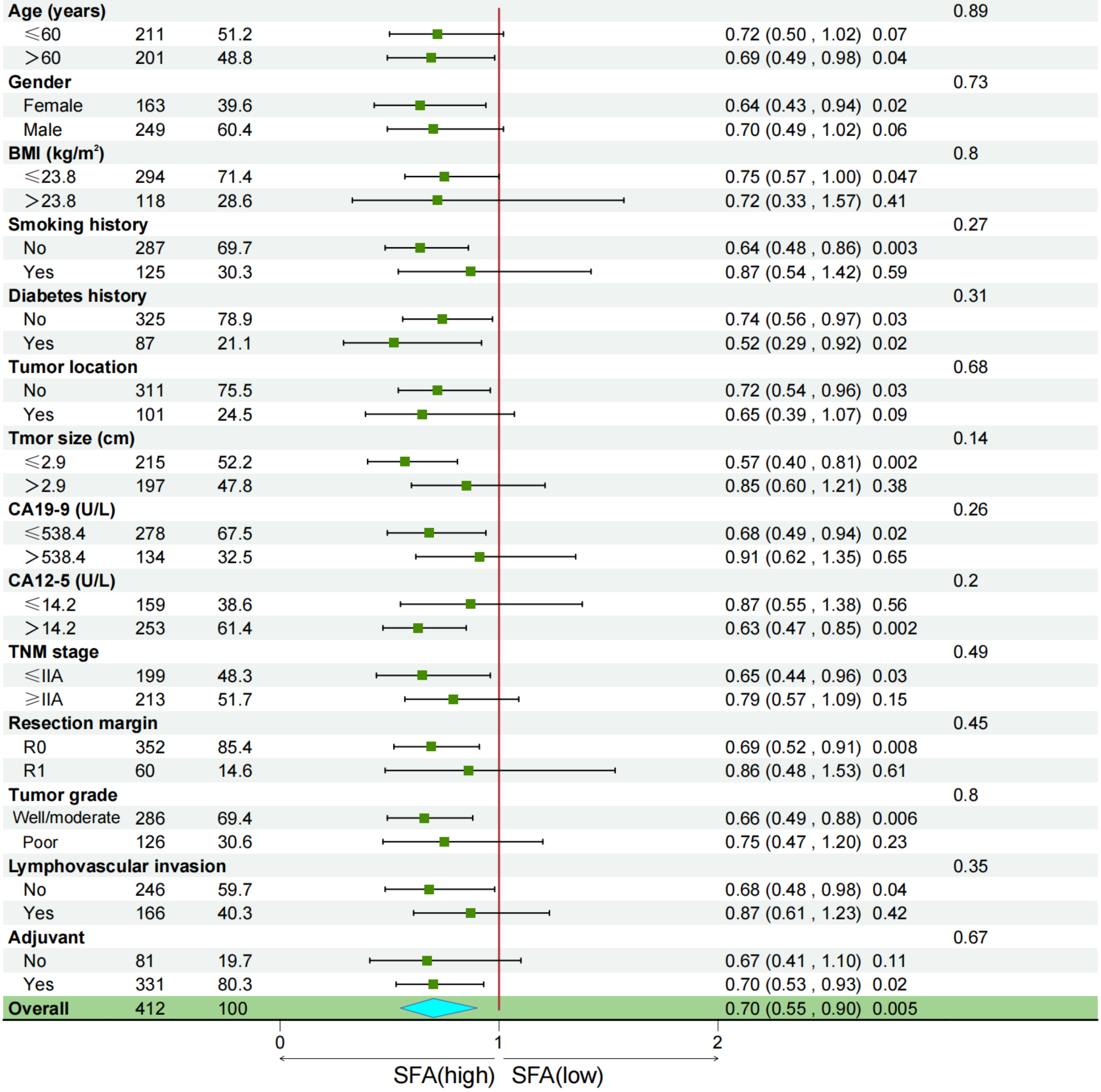

Figure S7. Forest plots showing stratified associations between SFA and overall survival of PDAC in the training set. *BMI: body mass index; CA19-9: carbohydrate antigen 19-9; CA12-5: carbohydrate antigen 12-5; PDAC: pancreatic ductal adenocarcinoma; R0: negative surgical margin, R1: positive surgical margin; SFA: subcutaneous fat area. TNM: tumor-node-metastasis.*

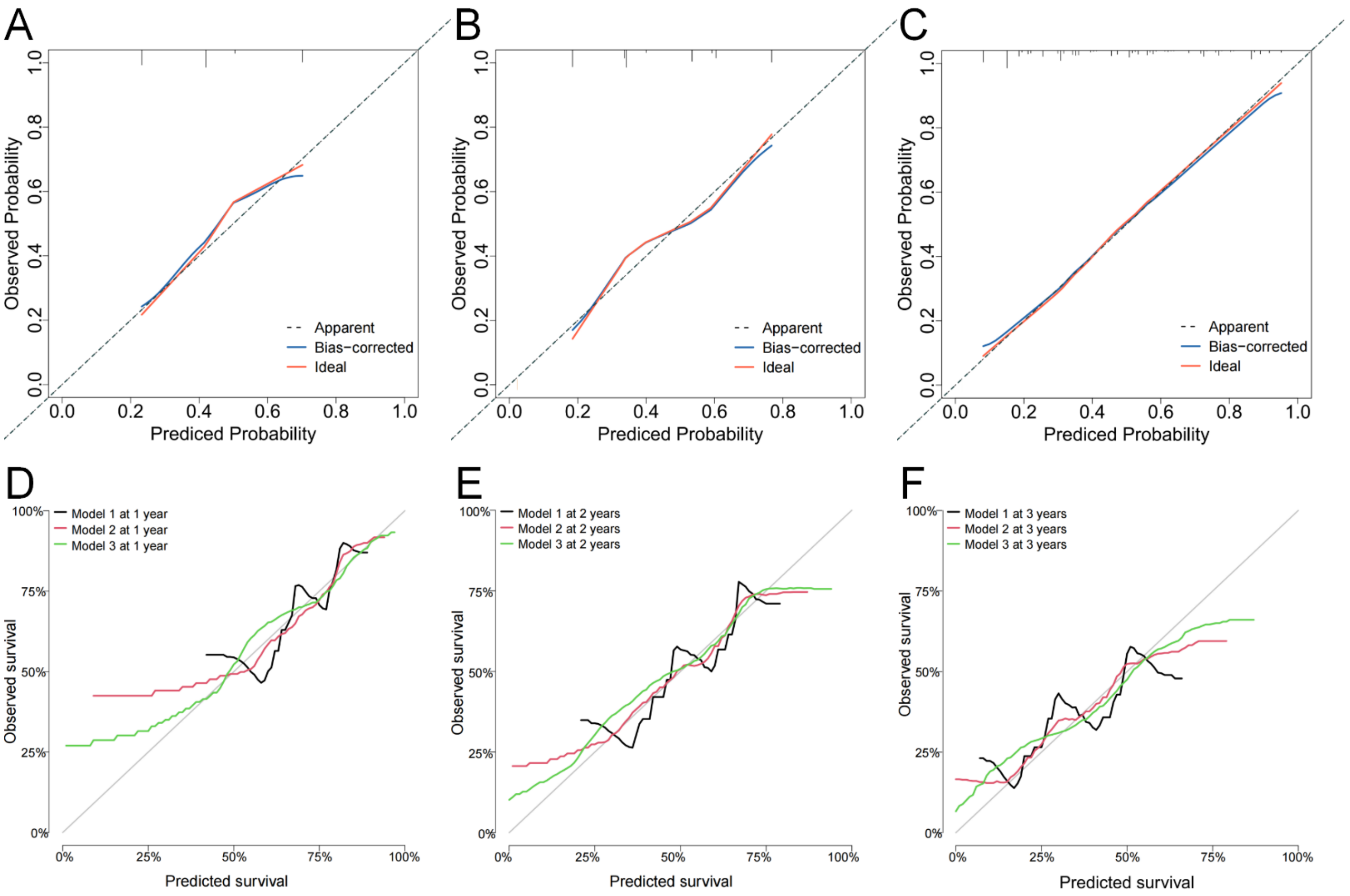

Figure S8. Calibration curves of the prediction models for early recurrence and overall survival in the training set. AUCs of the **A.** model 1 (CA19-9 and CA12-5), **B.** model 2 (CA19-9 and CA12-5 and VSR), and **C.** model 3 (CA19-9, CA12-5, VSR, TNM stage, lymphovascular invasion, and adjuvant therapy) for predicting early recurrence. **D-F.** AUCs of the models for predicting overall survival (Model1: CA19-9 and CA12-5; Model 2: CA19-9 and CA12-5, SFA, SMD, and VSR; Model 3: CA19-9 and CA12-5, SFA, SMD, VSR, TNM stage, lymphovascular invasion, and adjuvant therapy). *AUC, area under the ROC curve; SFA, subcutaneous fat area, SMD, skeletalmuscle density, TNM, tumour-node-metastasis, VSR, VFA-to-SFA.*

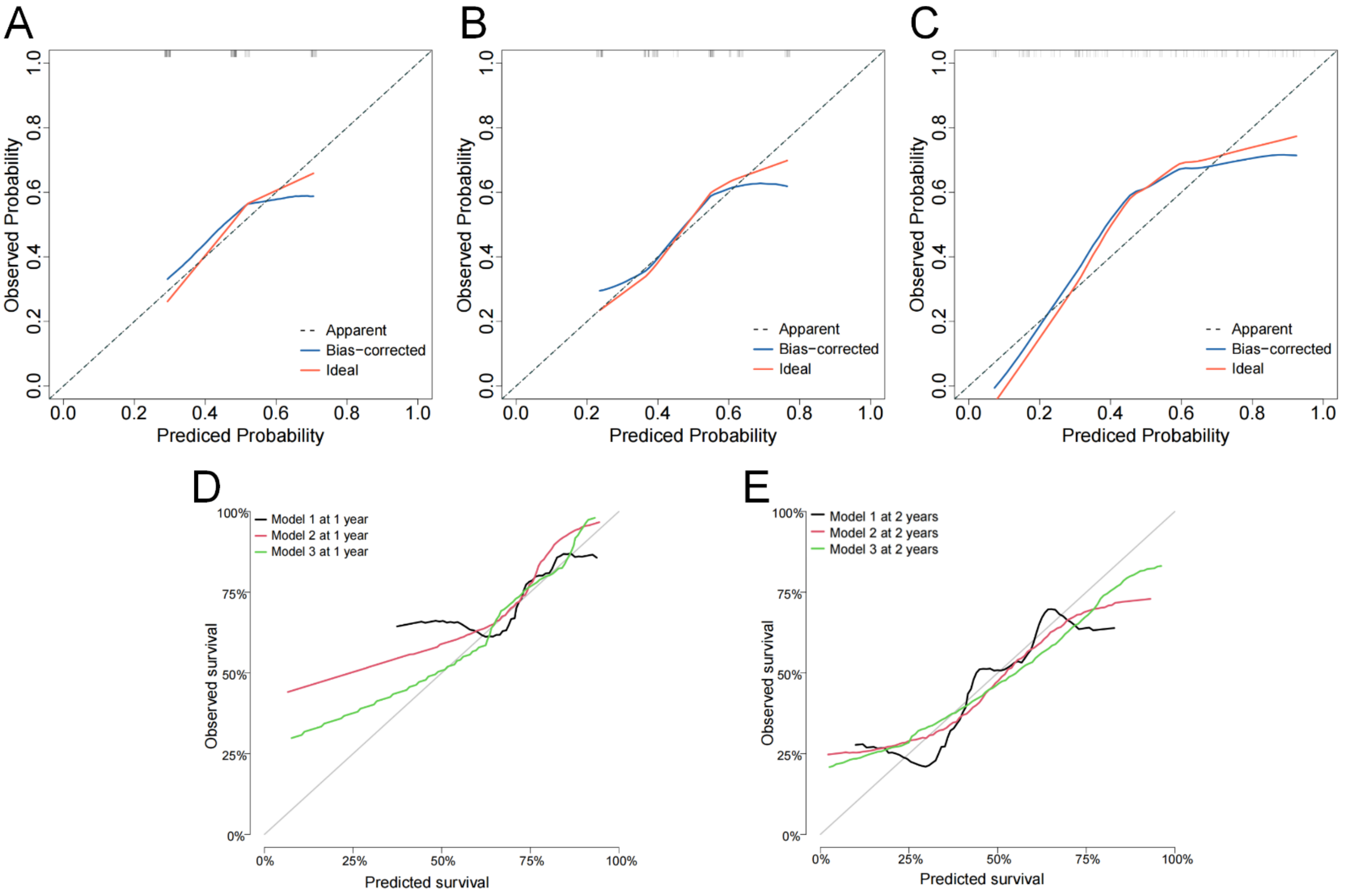

Figure S9. Calibration curves of the prediction models for early recurrence and overall survival in the external validation set. AUCs of the **A.** model 1 (CA19-9 and CA12-5), **B.** model 2 (CA19-9 and CA12-5 and VSR), and **C.** model 3 (CA19-9, CA12-5, VSR, TNM stage, lymphovascular invasion, and adjuvant therapy) for predicting early recurrence. **D-E.** AUCs of the models for predicting overall survival (Model1: CA19-9 and CA12-5; Model 2: CA19-9 and CA12-5, SFA, SMD, and VSR; Model 3: CA19-9 and CA12-5, SFA, SMD, VSR, TNM stage, lymphovascular invasion, and adjuvant therapy). *AUC, area under the ROC curve; SFA, subcutaneous fat area, SMD, skeletalmuscle density, TNM, tumour-node-metastasis, VSR, VFA-to-SFA.*
